# One-month physicochemical stability study of idarubicin prepared in polyolefin-type bags for the treatment of hematological diseases

**DOI:** 10.1371/journal.pone.0356906

**Published:** 2026-08-28

**Authors:** Guillaume Bouguéon, Arthur Jouvien, Jean-Marc Bernadou, Arnaud Venet, Aude Berroneau, Pierre Mora, Fabien Xuereb

**Affiliations:** 1 University Hospital of Bordeaux, Health Products Department, Hospital Pharmacy Unit, Pessac, France; 2 University Bordeaux, Inserm, ARNA (RNA: Natural and Artificial Regulations), ChemBioPharm, Bordeaux, France; 3 University Bordeaux, INSERM, BMC, Pessac, France; Assistance Publique - Hôpitaux de Marseille, FRANCE

## Abstract

Idarubicine is a mainstay of the treatment strategy for hematological cancer. The literature lacks stability data that comply with current preparation practices and recommendations for conducting stability studies. The aim of the present work is to establish the stability of idarubicin preparations diluted in polyolefin-type bags (Freeflex + ®) for intravenous administration in hematological clinical settings. Preparations were produced by diluting Zavedos® idarubicin in Freeflex + ® bags containing 0.9% sodium chloride (NaCl) or 5% dextrose (Dex) at concentrations of 0.04 mg/mL and 0.2 mg/mL. These preparations were stored at 5 ± 3 °C or 22 ± 3 °C. Three bags were prepared to evaluate each condition, for a total of 24 bags. To assess chemical stability, a stability-indicating high pressure liquid chromatography-ultraviolet/visible diode array detector assay was used. Samples were assayed on days D0, D1, D2, D7, D15, D22 and D30. A physical stability study was carried out on days D0, D15 and D30, looking for visible and sub-visible particles, measuring pH and osmolality. During the study, sample concentrations remained above 95% of the initial concentration, and no degradation products were detected. No color change or precipitation were observed with the naked eye. Sub-visible particles count remained below recommended limits. The pH and osmolality values remained unchanged from D0. This study demonstrates the stability of idarubicin, diluted to concentrations between 0.04 and 0.2 mg/mL in solutions of 0.9% NaCl and 5% Dex, stored at 5 ± 3 °C or 22 ± 3 °C for 30 days.

## Introduction

Idarubicin, a semi-synthetic anthracycline (S1 Fig), remains a mainstay of the treatment strategy for newly-diagnosed acute myeloblastic leukemia in children and adults, or for patients with relapsed or refractory disease, ahead of other anthracyclines. Idarubicin is also used in the treatment of recurrent acute lymphoblastic leukemia in children and adults [1–3]. For solid tumors, idarubicin is used in gastroenterology in transarterial chemoembolization (TACE) therapies mixed with contrast medium emulsions (e.g., Lipiodol®) for the treatment of intermediate-stage liver carcinoma [4].

According to the current guidelines, idarubicin is used in the treatment of acute leukemia in children and adults at dosages ranging from 5 to 12 mg/m^2^ administered daily intravenously in regimens lasting 3–5 days. For administration to patients, idarubicin must be diluted in bags or syringes with 0.9% sodium chloride (NaCl) or 5% Dextrose (Dex) [1]. Given that solvent bag dilution is commonly used in the treatment of hematological diseases, this study will focus on this preparation method, syringe dilution being used in a chemoembolization context.

In centers specialized in hematology and providing care for a large number of patients, the number of idarubicin preparations needed can be high, requiring advance preparation for organizational and logistical reasons. Hospital pharmacists responsible for producing sterile cytotoxic preparations, in accordance with applicable good preparation practices, must therefore have reliable data on the stability of these preparations after dilution in solvents, beyond the 24 hours recommended by pharmaceutical firms [1,5–7].

Regarding the dilution of idarubicin in infusion bags, the only study available was conducted by Beijnen et al. in 1985 [8]. In this article, the stability of idarubicin diluted to a concentration of 0.1 mg/mL in 0.3% and 0.9% NaCl, 3.3% and 5% Dex and Ringer Lactate was assessed. The study was conducted at room temperature and samples were protected from light. Beijnen *et al*. concluded that idarubicin was stable for a period of 28 days. However, this work has limitations and lacks specific details regarding the criteria currently recommended for stability study procedures [6,9,10]. Indeed, this study was not conducted under routine clinical conditions of use, idarubicin having been diluted in polypropylene tubes and not in bags of diluents for infusion. Furthermore, fundamental criteria on physicochemical stability (determination of visible, sub-visible particles, osmolality, and pH monitoring) are missing or lack details. Moreover, idarubicin concentrations used in routine practice frequently deviate from the target value of 0.1 mg/mL studied or may be stored in the refrigerator by accident. Extrapolating data from stability studies to conditions other than those studied (e.g., concentrations, solvent, storage conditions) involves risks and engage the responsibility of the pharmacists who produce these preparations [6,9,10].

For these reasons, we propose to study the stability of idarubicin preparations under conditions of use in line with current practices: dilution in polyolefin-type bags (Freeflex + ®) in 0.9% NaCl and 5% Dex, at concentrations between 0.04 mg/mL and 0.2 mg/mL, stored at room temperature or refrigerated, and protected from UV.

## Materials and methods

### Materials

Idarubicin 10 mg/10 mL (Zavedos®, Pfizer, New York City, NY, US) (batches CA7906 exp: 2022 03 and DX0725 exp: 2023 03) was used to prepare idarubicin bags of 100 mL 0.9% sodium chloride (NaCl) and 100 mL 5% dextrose (Dex). For solution dilution, Freeflex + ® bags were purchased from Fresenius Kabi (Bad Homburg vor der Höhe, Germany). The infusion bags were made of polyolefin (primary internal film: polypropylene; secondary external film: polypropylene/polyethylene admixture).

The following materials were used for the analytical experiments: idarubicin hydrochloride powder (Sigma Aldrich (Merck KGaA, Darmstadt, Germany)(batch 0000424922 exp: 2024 11), distilled water provided in-site with a Milli-Q® de-ionization system (PURELAB® Flex 1, ELGA CA, High Wycombe, UK), acetonitrile (ACN) HPLC-S gradient grade (Biosolve, Deuze, France), potassium dihydrogen phosphate (KH_2_PO_4_) (Prolab, Sion, Switzerland), 30% hydrogen peroxide (H_2_O_2_) (Cooper, Melun, France), AnalaR Normapur 37% hydrochloric acid (HCl) and 30% sodium hydroxide (NaOH) (VWR Chemicals Avantor, Radnor, PA, US).

### Choice of concentrations for the stability study

To guide the choices of concentrations for the stability study, a preliminary analysis of injectable anticancer drug production data from University Hospital of Bordeaux was conducted. The data were extracted from Chimio® production management software (Computer Engineering, Paris, France) and were centered on idarubicin production in bags between 2016 and 2023 intended for adult and pediatric oncohematology care units.

University Hospital of Bordeaux makes approximately 80,000 injectable preparations per year. Over the study period, 2,172 idarubicin bags were made as part of acute lymphoid leukemia, acute myeloid leukemia and lymphoma treatment regimens (S8 Fig). Preparations intended for adults represented over 95% of production. As the dose banding approach was not applied for idarubicin dose standardization, 23 different concentrations between 0.02 and 0.28 mg/mL were produced, of which close to 87% were centered between 0.09 and 0.15 mg/mL. The limits of the stability study were defined between 0.04 mg/mL and 0.2 mg/mL, with concentrations beyond these limits concerning fewer than 10 preparations over the 8 years of analysis.

### Idarubicin bag preparation

Idarubicin bags were prepared from the brand-name drug Zavedos® idarubicin, 10 mg/10 mL and diluted in 100 mL polyolefin-type infusion bags (Freeflex + ®). Three variation factors were studied and crossed: the diluent (0.9% NaCl or 5% Dex), the final concentration (0.04 mg/mL or 0.2 mg/mL) and the storage temperature (room temperature 22 ± 3 °C or refrigerated conditions 5 ± 3 °C). In this way, eight conditions were studied. For each condition, the bags were prepared in triplicate, for a total of 24 bags. All the preparations were produced under aseptic conditions in an ISO 5 (International Organization for Standardization) isolator at negative pressure (JCE Biotechnology, Hauterive, France). The preparations were then stored protected from UV in a photoprotective secondary packaging (200300UVS+ (SLB, Genas, France)).

### Collective and individual protective measures against cytotoxic risks

All staff involved in sample preparation and quality control during the stability study have received training and are authorized to handle cytotoxic medicines. The preparation of cytotoxic drugs was performed in an ISO 5 negative-pressure isolator located within an ISO 7 controlled-atmosphere area. Operators wore appropriate protective clothing for work in these areas: reusable fabric scrubs and gowns, single-use caps, and single-use non-sterile gloves. During the control steps, sample preparation was performed in areas with an unclassified atmosphere. Appropriate collective protective measures were taken: handling under a microbiological safety cabinet class 2, use of sterile drapes. Technicians wore appropriate protective clothing: non-sterile gloves, a single-use lab coat, and a single-use mask.

### Stability-indicating high pressure liquid chromatography-ultraviolet/visible diode array detector (HPLC-UV/VIS DAD) assay method

The idarubicin assay was conducted with a high-performance liquid chromatographic method adapted from the method described by Kaushik *et al* [11]. The apparatus used was a Thermo Scientific^TM^ UltiMate^TM^ 3000 HPLC-UV/VIS system, coupled with a photodiode array detector (DAD, diode array detector) (Thermo Fischer, MA, US). Separation was conducted on an XDB-C18 column (250 mm x 4.6 mm; particle size 5 µm) (Agilent, Santa Clara, CA, US) preceded by a 0.5 µm frit (Thermo Fischer Scientific, Waltham, MA, US). The mobile phase was a 60:40 mixture composed of a 10 mM pH 2.8 KH_2_PO_4_ phosphate buffer and acetonitrile. The analyses were conducted under isocratic elution conditions (1 mL/min) at a temperature of 25 °C. Samples awaiting analysis were stored in the autosampler at a temperature of 4 °C. The volume of sample injected was 5 µL. Data were acquired at 254 nm. Data acquisition and peak purity match were performed using Thermo Scientific^TM^ Chromeleon^TM^ software Version 7.2.7 (Thermo Fischer Scientific, Waltham, MA, US).

### HPLC-UV/VIS DAD assay method validation

The samples used to validate the assay method were prepared each day, extemporaneously from idarubicin hydrochloride powder (purity 100%, water content 2.1%) diluted in distilled water provided in-site with a Milli-Q® de-ionization system (PURELAB® Flex 1, ELGA CA, High Wycombe, UK). The validation of the assay method was conducted in accordance with international conference on harmonization (ICH) Q2 (R2) guidelines [12].

The linearity of the method was assessed by producing a serie of five-point concentrations (0.010, 0.025, 0.050, 0.100 and 0.250 mg/mL) from independent test samples. This serie was repeated on 3 different days. The linear regression analysis was conducted using Chromeleon^TM^ software. The method was considered linear if all the correlation coefficients (R^2^) calculated were greater than 0.99. The accuracy (expressed as %) and the precision (expressed as a coefficient of variation CV%) were assessed through the intraday and interday repeatability, using 3 quality controls prepared at concentrations of 0.02, 0.15 and 0.2 mg/mL. The intraday repeatability (precision and accuracy) was assessed using the three quality control points, each prepared and analyzed 6 times. The interday repeatability (precision and accuracy) was determined by repeating the same assay on 3 different days. The accuracy was considered acceptable if the values obtained were between 98 and 102%. The precision was considered acceptable if the CV values obtained were not greater than 2%. The detection limit was determined from the background noise and was considered to correspond to the concentration at which the signal to noise ratio was 3:1. The quantitation limit was determined based on a signal to noise ratio of 10:1.

### Forced degradation study

In order to be able to detect any degradation products, the ability of the assay method to be stability-indicating was assessed according to ICH Q1A(R2) and SFPC/ GERPAC guidelines [9,10]. To this end, one-mL samples prepared from idarubicin hydrochloride powder were exposed to different forced degradation conditions. For the heating stress conditions, one mL of idarubicin was heated for 4 hours at 80 °C in a Jouan water bath (Thermo Electron, ThermoFischer Scientific, Nantes, France) and analyzed undiluted. For oxidative stress conditions, one mL of idarubicin was mixed with one mL of 7.5% (w/w) hydrogen peroxide (H_2_O_2_) for 4 hours (sample diluted 1:2). For acidic stress conditions, one mL of idarubicin was mixed with one mL of a hydrochloric acid (HCl) solution 0.1 M and heated to 80 °C in a water bath for 5 min; before analysis, the samples were neutralized with one mL of sodium hydroxide solution (NaOH) 0.1 M (sample diluted to one-third). For alkaline stress conditions, one mL of idarubicin was mixed with one mL of a sodium hydroxide (NaOH) solution 1 M and heated to 80 °C in a water bath for 1 min; before analysis, the samples were neutralized with one mL of hydrochloric acid solution (HCl) 1 M (sample diluted to one-third). For photolytic stress conditions, four mL samples of idarubicin were exposed to a fluorescent lamp emitting 29,000 lux at 15 cm and at room temperature of 22 ± 3 °C for 2 hours (Sylvania Sylfast SSE T5, Osram Sylvania, Munich, Germany) and analyzed undiluted. All the chromatograms obtained were compared to the reference chromatograms obtained with the assay method described above, using samples not subjected to stress conditions. The degradation parameters were optimized to ensure effective separation down to the baseline, or to minimize overlap between the peaks of the degradation products and the idarubicin peak. All tests were performed in triplicate.

### Stability study design and acceptance criteria

The chemical stability of the idarubicin preparations over time, under the 8 study conditions, was determined by assessing the idarubicin concentration on 7 study days: D0, D1, D2, D7, D15, D22 and D30. Each day, three bags of each condition were analyzed, and the mean concentration and standard deviation calculated. The preparations were considered stable if the individual concentrations measured at the different time points during the study were within the 90–110% interval of the initial concentration (D0) [9].

### pH determination

The pH was determined using a Seven Compact^TM^ pH-meter equipped with an Inlab® Micro-Pro-ISM 51344163 probe (Mettler Toledo, Colombus, Ohio, US). Three-point calibration were conducted with the pH 4.01, 7.00 and 9.21 buffer solutions available from Mettler Toledo. The assays were conducted on 2 mL samples in triplicate. Throughout the study, it was verified that the pH variation did not exceed one unit.

### Osmolality determination

The osmolality was measured using a Type 15 cryoscopic osmometer (Löser Messtechnik, Berlin, Germany). Calibration was conducted at 0, 300 and 900 mOsm/kg using water for injections (point 0 mOsm/kg, PROAMP, Aguettant, Lyon, France) and reference solutions available from Löser Messtechnik. The measurements were conducted on 100 µL samples in triplicate.

### Sub-visible particles count

The sub-visible particles assay was performed in accordance with the European Pharmacopoeia (Test 2.9.19 Particulate contamination: sub-visible particles, test 1.B) and was conducted using an HIAC 9703 + particle counter coupled with an HRLD 150 sensor (Beckman Coulter, Brea, California, US). The sample volume used was 25 mL. For each sample, the apparatus calculated a mean of 4 successive measurements. Samples were compliant if the number of the average number of particles present in each bag tested does not exceed 6,000 particles of ≥10 μm or 600 particles of ≥25 μm in accordance with the European Pharmacopoeia [13].

### Organoleptic inspection

The idarubicin bags were observed visually on a STETDMLED12 visual inspection table (STERIGEN, Upton, Canada), alternately for 5 seconds in front of a white panel followed by 5 seconds in front of a black panel to detect visible particles or a change of color. The comparison was made with a preparation produced extemporaneously on the day of observation [9,13]

## Results

### Assay method validation

**Fig 1:**
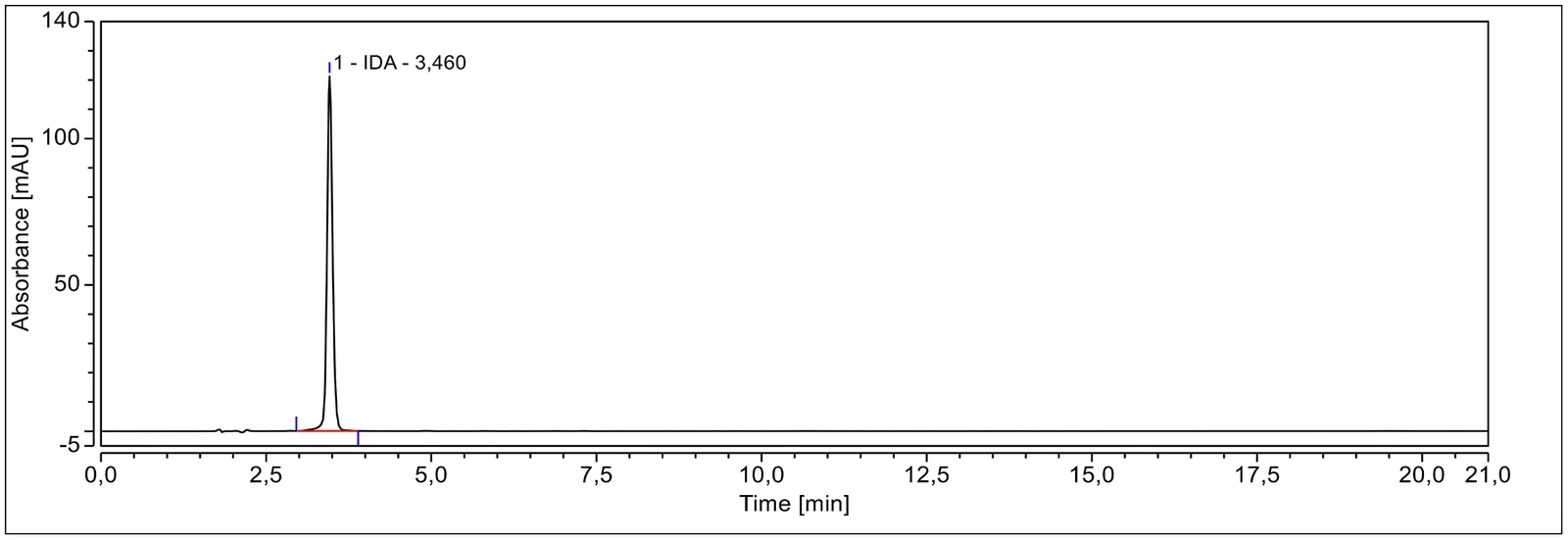
Idarubicin 0.04 mg/mL reference chromatogram from the chromatographic stability indicating method developed to detect degradation products (total analysis time: 22 min). Abbreviation: IDA: idarubicin.

The chromatograms obtained showed a well-defined and symmetrical peak with a mean retention time of 3.4 min and baseline only exhibiting a low level of background noise (Fig 1). The calibration curves produced on 3 different days, were linear with a correlation coefficient (R^2^) of 0.999. Both the intraday and interday repeatability have produced satisfactory results (Table 1). The intraday and interday accuracies ranged between 98.09 and 101.73% and between 99.45 and 101.24% respectively. Similarly, the CV% values ranged between 0.04 and 0.37% and between 0.41 and 1.47% respectively. The limit of detection (LOD) was assessed at 6.01 x 10^−5^ mg/mL and the limit of quantification (LOQ) at 20.03 x 10^−5^ mg/mL. The HPLC-UV/VIS DAD assay method complies with ICH Q2 (R2) requirements [12].

**Table 1:** Intra and interday validation of the chromatographic method.

	Days	Theoretical concentration (mg/mL)	Observed concentration (mg/mL) (mean ± S.D.)	Accuracy %	Precision CV%	n	Linearity y= ax+b
Intraday	1	0.0200	0.0196 ± 0.0000	98.09	0.12	6	a = 314.729 b = 0.076 R^2^ = 0.999
		0.1500	0.1484 ± 0.0001	98.93	0.04	6	
		0.2000	0.2035 ± 0.0002	101.73	0.09	6	
	2	0.0200	0.0203 ± 0.0000	101.44	0.22	6	a = 311.769 b = 0.058 R^2^ = 0.999
		0.1500	0.1506 ± 0.0003	100.40	0.17	6	
		0.2000	0.2016 ± 0.0004	100.82	0.20	6	
	3	0.0200	0.0198 ± 0.0001	98.83	0.37	6	a = 316.665 b = −0.090 R^2^ = 0.999
		0.1500	0.1491 ± 0.0002	99.42	0.12	6	
		0.2000	0.2023 ± 0.0003	101.13	0.13	6	
Interday	Over 3 days	0.0200	0.0199 ± 0.0003	99.45	1.47	18	
		0.1500	0.1494 ± 0.0009	99.58	0.62	18	
		0.2000	0.2025 ± 0.0008	101.24	0.41	18	

Abbreviations: S.D.: standard deviation; CV: coefficient of variation

### Stability-indicating method

Regarding the forced degradation study of idarubicin, different degradation levels were obtained according to the exposure conditions (Table 2). The chromatograms were compared to the reference chromatogram illustrated by Fig 1.

**Table 2:** Forced degradation study results.

Type of degradation	Conditions	Percentage of degradation	Mass balance (%)
Heating stress	80 °C	38.2% 4 h	60.8
Oxidative	H_2_0_2_ 7.5%	22.7% − 30 min	89.1
Acidic	HCl 0.1 M – 80 °C	34.3% − 5 min	95.2
Alkaline	NaOH 1 M – 70 °C	15.2% − 1 min	86.8
Photolytic stress	UV – 20 °C	19.3% - 2h	87.1

Under heating conditions, the degradation of idarubicin was calculated at 38.2% and the chromatogram obtained detected degradation products at the retention times of 3.98, 4.48 and 7.32 min annotated as DEG H1, DEG H2 and DEG H3 respectively (S2 Fig).

Under oxidative conditions, the degradation of idarubicin obtained was 22,7% % and one degradation product was detected at 2.47 min annotated as DEG OX1 (S3 Fig).

The sensitivity of idarubicin to acidic stress conditions showed degradation levels of 34.3%. However, under these conditions, few changes in the chromatogram compared with the reference chromatogram were observed (S4 Fig). Only one peak detected at the end of the analysis with a retention time of 19.7 min was observable (DEG AC1).

However, idarubicin was more sensitive to degradation under alkaline stress conditions. In fact, after just one minute of contact, a 15% degradation is observed, with at least four identifiable peaks (DEG ALK1 to DEG ALK4) between 3.11 and 4.87 min (S5 Fig).

Idarubicin showed a high sensitivity to degradation when exposed to light. Nearly 20% of the initial product was degraded within 2h. Seven degradation products were recorded (DEG L1-DEG L7) between 1.79 and 6.92 min (S6 Fig).

The assay method used enabled the detection of idarubicin degradation products under all the study conditions. In accordance with the guidelines, the degradation of the idarubicin peak was close to 20% under each condition compared to the reference peak [9]. Furthermore, the purity of the residual idarubicin peak was systematically greater than 99.5%. For these reasons, the assay method used was validated as a stability-indicating method.

### Physicochemical stability

Over the 30 days of the physicochemical stability study, three variation factors were studied: concentration, diluent, and storage temperature. Whatever the study conditions, the HPLC-UV/VIS DAD assay results showed the stability of the preparations. The mean idarubicin concentrations remained above 95% (from 97.43 to 102.4%) of the initial concentrations for the 24 bags tested (Table 3, S7 Fig). Furthermore, no degradation products were observed, as all the chromatograms being similar to the reference chromatogram (Fig 1) at all seven analysis time points, and the purity of idarubicin peaks remained above 99.5%.

**Table 3:** Chemical stability study results of idarubicin diluted in polyolefin-type bags.

Concentration	0.04 mg/mL							
Diluent	0.9 % NaCl				5 % Dextrose			
Temperature	5 ± 3 °C		22 ± 3 °C		5 ± 3 °C		22 ± 3 °C	
Storage Time	Av	S.D.	Av	S.D.	Av	S.D.	Av	S.D.
D0	100.00	0.00	100.00	0.00	100.00	0.00	100.00	0.00
D1	98.04	2.64	98.48	1.15	97.43	1.87	97.86	1.56
D2	98.04	2.59	98.48	0.98	97.81	0.98	101.07	1.72
D7	101.03	0.89	100.19	2.12	101.86	2.28	101.75	1.87
D15	99.14	2.80	100.24	0.24	98.44	1.67	99.88	1.24
D22	102.39	0.75	100.30	1.98	97.71	1.07	100.08	0.96
D30	98.38	1.33	100.79	1.24	98.65	1.06	100.80	1.74
**Concentration**	**0.2 mg/mL**							
**Diluent**	**0.9 % NaCl**				**5 % Dextrose**			
**Temperature**	**5 ± 3 °C**		**22 ± 3 °C**		**5 ± 3 °C**		**22 ± 3 °C**	
**Storage Time**	**Av**	**S.D.**	**Av**	**S.D.**	**Av**	**S.D.**	**Av**	**S.D.**
D0	100.00	0.00	100.00	0.00	100.00	0.00	100.00	0.00
D1	99.05	0.17	99.26	0.15	98.94	0.52	98.32	0.27
D2	99.05	0.46	99.26	0.19	99.21	0.42	99.73	0.67
D7	100.89	0.26	101.28	0.50	100.83	0.34	101.52	0.42
D15	100.26	0.43	100.46	0.56	99.41	0.83	99.56	0.58
D22	101.84	0.27	102.40	0.36	101.60	0.77	100.94	0.68
D30	101.08	0.39	101.64	0.14	101.03	0.87	100.23	0.49

Abbreviations: NaCl: sodium chloride; Av: average; S.D.: standard deviation; D: Day

**Fig 2:**
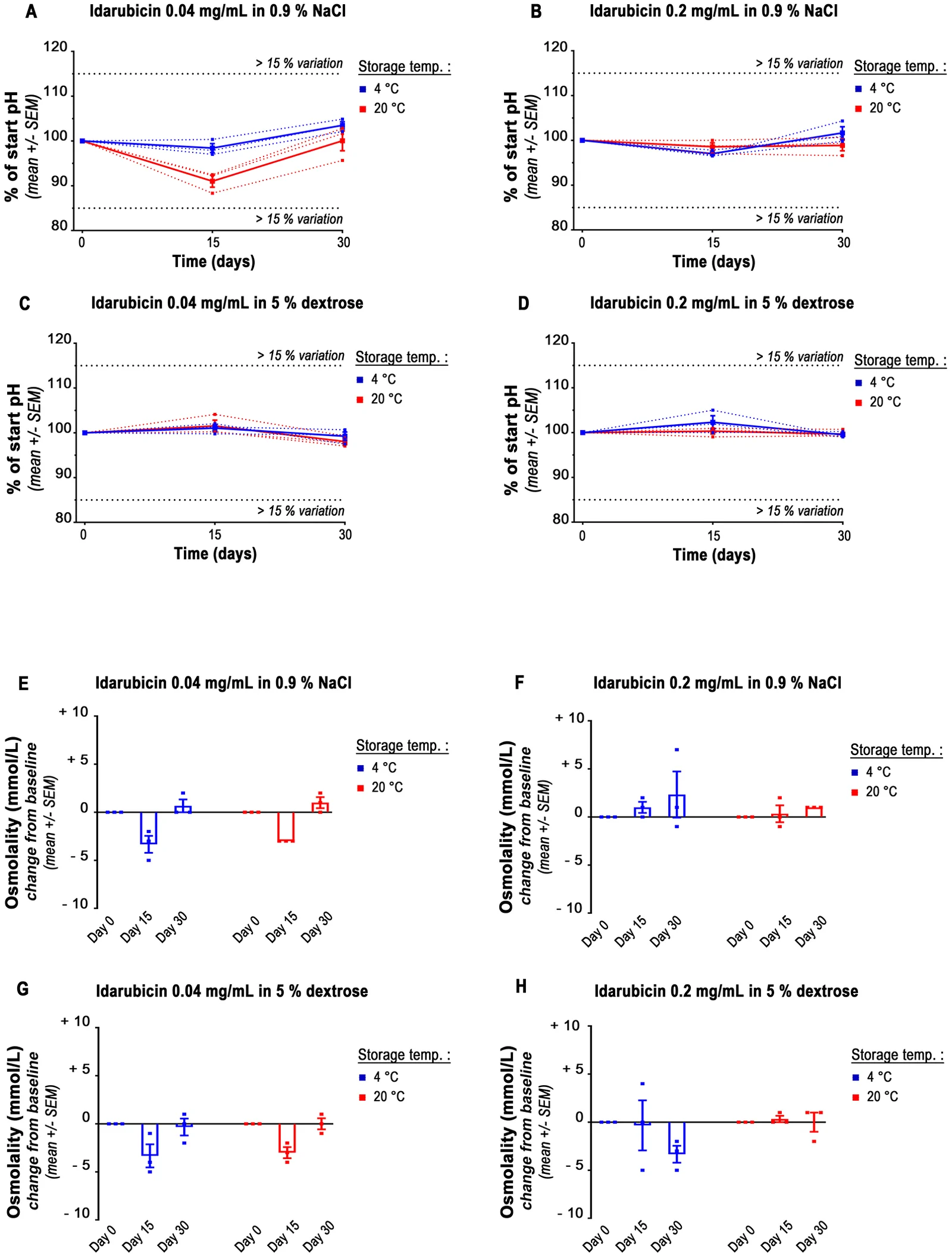
Measurements of pH (A, B, C, D) and osmolarity (E, F,) over time of idarubicin diluted at 0.04 mg/mL and 0.2 mg/mL in 0.9% NaCl and 5% Dex IV bags. Abbreviations: Conc: concentration; NaCl: sodium chloride; Dex: dextrose; SEM: standard error of mean; Temp: temperature.

For the pH measurements on days D0, D15 and D30, in the 0.9% NaCl and 5% Dex bags, no variation greater than one pH unit was observed compared to the pH at the start of the study (D0) (S1 Table, Fig 2) independently of temperature.

Similarly, osmolality of the 0.9% NaCl and 5% Dex bags on days D0, D15 and D30 remained stable at around 5 units on average compared with the osmolality on D0 (S2 Table, Fig 2).

**Fig 3:**
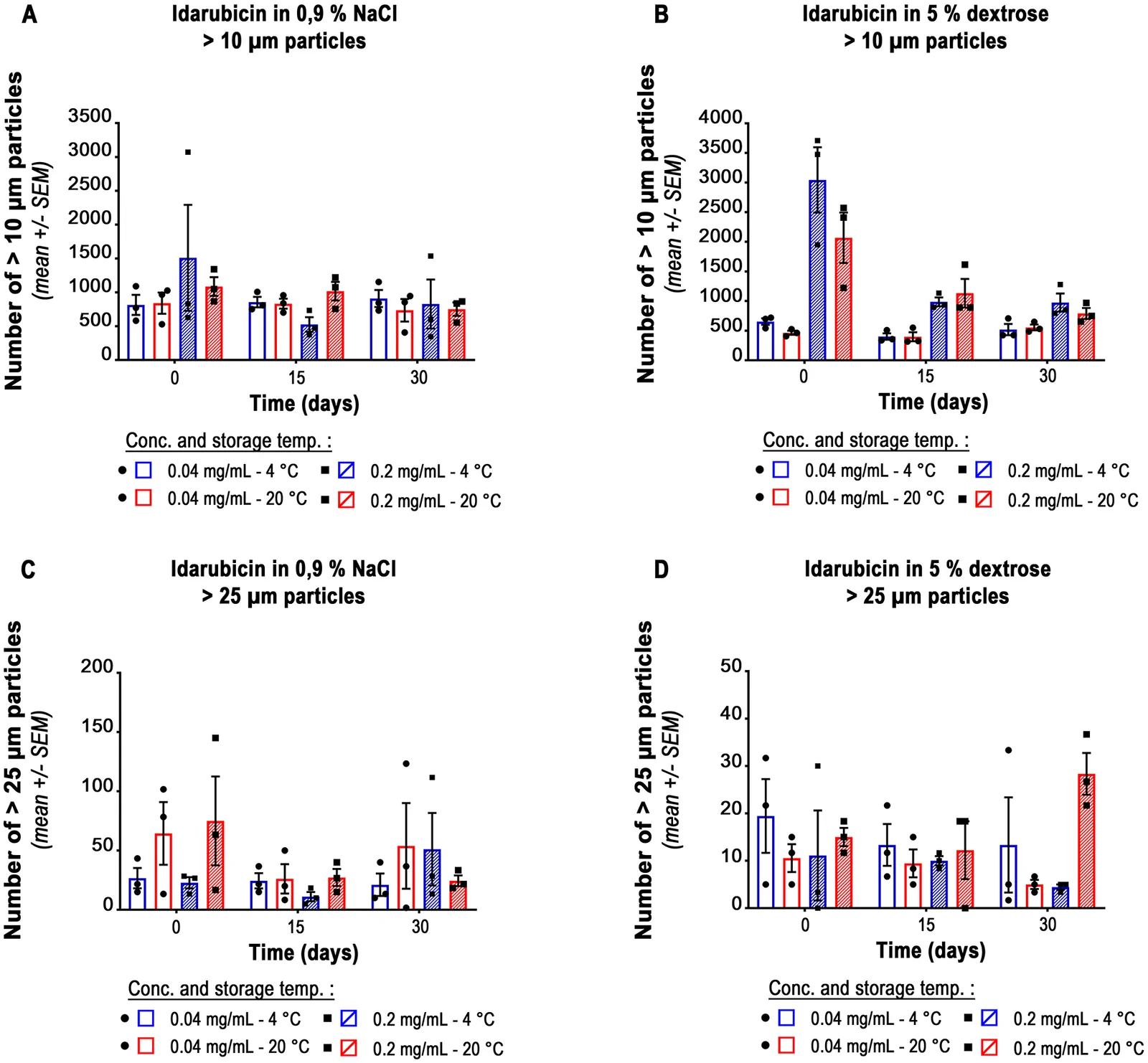
Sub-visible particles of >10 µm (A, B) and of <25 µm (C, D) measurement over time of idarubicin diluted at 0.04 mg/mL and 0.2 mg/mL in 0.9% NaCl and 5% Dex IV bags. Abbreviations: Conc: concentration; NaCl: sodium chloride; Dex: dextrose; SEM: standard error of mean; temp: temperature.

For the particle count on days D0, D15 and D30, the stability study demonstrated compliance with European Pharmacopoeia guidelines in both 0.9% NaCl and 5% Dex bags. The number of particles measured remained below 6,000 per container for particle ≥10 µm in size of and below 600 per container for particle ≥25 µm in size (S3 Table; Fig 3) [13].

The visual inspection did not show the presence of precipitates or particles. Furthermore, no change of color was observed with the naked eye.

## Discussion

The present study focused on the brand-name drug Zavedos®, the most important idarubicin presentation on the market, in ready-to-use form, in which the excipients are glycerol, hydrochloric acid (QS pH from 3 to 4) and water for injections. It was conducted on Freeflex + ® infusion bags made of polyolefins.

We did not demonstrate any influence of the concentration, storage conditions or diluent on physicochemical stability. The results remained above 95% without observing any degradation products. The pH and osmolality variations were acceptable. The pH observed in our preparations remained closer to the bag pH limits indicated by the supplier (pH of the brand-name drug Zavedos® undiluted: from 3 to 4; pH of solvent bags: from 4.5 to 7.0 for 0.9% NaCl and from 3.5 to 6.5 for 5% Dex).

Finally, with regard to the presence of particles, counts remained below the recommended limits. However, at D0, a dispersion of the results was observed with a higher number of particles than on the other two study days, particularly for particles of >10 µm. We assume that the particle count was carried out quickly after preparation of the bags and that dilution of the idarubicin may not have been complete. This could also be explained by contamination of the measuring device (which was not located in a controlled atmosphere area) or contamination of the glass vials into which the samples were transferred for counting by the instrument. For this reason, during the subsequent procedures, particular attention was paid to the movements around the analyser and to the preparation of the samples prior to analysis.

To date, the only stability study available in the scientific literature on the stability of idarubicin preparations in bags is that of Beijnen *et al.* performed with a non-stability-indicating HPLC method [8]. This study presents the stability data for doxorubicin and five of its derivatives in a wide range of solvents (0.3% and 0.9% NaCl, 3.3% and 5% Dex and Ringer Lactate) and suggests that idarubicin is stable for 28 days at 0.1 mg/ml.

The results of the present study are consistent with these data and update them by exploring notably the stability of idarubicin in refrigerated conditions and above 0.1 mg /mL. The selected concentrations study chosen were based directly on clinical practice and thus cover a broad concentration range (S8 Fig). Our review of clinical practices showed that the most commonly used concentrations ranged from 0.09 to 0.15 mg/mL with a mean of 0.12 mg/mL (from 0.057 to 0.185 mg/mL). The outermost concentrations, representing less than 1% of activity, have not been taken into account as they are too infrequent. For this reason, the concentration limits of 0.04 to 0.2 mg/mL were selected for the study.

Despite the qualities of the Beijnen *et al.* study, it did not meet current criteria for stability studies [6,9,10]. Indeed, the study was simulated in polypropylene tubes and not in infusion bags from which samples would have been taken over time. This raises questions about the possibility of interpreting these data and transposing them to real storage conditions in polyolefin-type bags. We cannot judge the validation of the chromatographic method used in this study or its ability to separate and detect any degradation products by a forced degradation study. In addition, fundamental criteria on physicochemical stability (determination of visible, sub-visible particles, osmolality, and pH monitoring) are missing or lack details. We have provided further details on these points.

In accordance with cytotoxic preparation stability study guidelines, the separation and analysis method (HPLC-UV/VIS DAD) that we used were validated. We were able to degrade the molecule and separate the pure peak of idarubicin from its degradation products which demonstrated the ability of the method to indicate stability [6,9]. In the context of the present study, the DAD detector used provided reliable, specific, and sufficient data on idarubicin stability and detection of degradation products, notably because we worked in a non-complex environment and at concentrations compatible with the DAD detector’s efficiency range. The DAD detector allowed the development of sufficiently sensitive methods, allowed the peak purity to be assessed and has the advantage of being accessible to small analysis facilities, particularly hospitals, which are not equipped with more sensitive methods such as mass spectrometry. Our chromatographic conditions were roughly similar to those of Kaushik *et al.* [11] and Bourcier *et al*. [14] although they both used UV coupled to mass spectrometry detectors. We observed the same degradation behavior of idarubicin and the same sensitivity to alkaline, acidic, and oxidative conditions. In addition, Bourcier *et al.* noticed a sensitivity to thermal and photolytic conditions, not described by Kaushik *et al.*, that we also confirmed [15].

In the present study, all factors that may influence the physical and chemical stability of injectable preparations (e.g., temperature, hydrolysis, redox reactions, photosensitivity, precipitation) have been tested [15]. The results of the accelerated degradation study show that the molecule is sensitive to extreme temperatures (80°C). Our study was not conducted at temperatures exceeding 25°C. We therefore recommend that transport and storage take place at controlled temperatures. Nevertheless, given the short duration of administration recommended by the suppliers (approximately ten minutes) [1], a temperature deviation of <35°C within this time frame should not significantly affect the stability of the molecule.

Idarubicin is sensitive to acidic (0.1N HCl) and extremely basic (1M NaOH) conditions. The various hydroxyl, amine and ketone groups, as well as the glycosidic bond and the aromatic ring, may therefore undergo significant chemical reactions under these conditions, leading to the formation of degradation products. The pH monitoring showed an acceptable variation around the values measured at Day 0 and did not reveal any significant formation of H+ or OH- ions over the study period. Furthermore, as no degradation products were observed within the detection limits of DAD spectroscopy, idarubicin was therefore not subject to significant hydrolysis.

When preparing the samples for the study, the residual air in the infusion bags was not removed. The accelerated degradation study demonstrated degradation under oxidative conditions caused by H₂O₂. In light of the results of the stability study, the residual air in the bags did not cause any oxidation or affect the stability of idarubicin. This air therefore does not need to be removed when preparing the infusion bags.

With regard to light sensitivity, the accelerated degradation study demonstrated the instability of the idarubicin molecule when exposed to UV radiation. For this reason, the study was conducted by protecting the infusion bags with light-protective packaging. We therefore recommend that users protect their bags. If the administration is brief, as recommended by the suppliers, the idarubicin will pass through an unprotected infusion tube quickly and is very likely to undergo minimal degradation. Should the administration take longer than 30 minutes for various reasons, we recommend using a light-protective infusion tube.

Finally, during our study, we did not observe any precipitates either with the naked eye or under the microscope. Similarly, no change in colour was observed. Nevertheless, should any change in colour or the appearance of a precipitate be observed under clinical conditions, administration of the product should be discontinued and further investigations carried out to determine the causes.

Idarubicin is an anthracycline; the main representatives of this class used in oncology are doxorubicin, epirubicin and daunorubicin. Idarubicin is an analogue of daunorubicin, differing in the absence of a methoxy group at the 4’ position. Doxorubicin differs from daunorubicin in that it has an additional hydroxyl group at the 14’ position. Epirubicin, meanwhile, is an epimer of doxorubicin at the C4’ position of the sugar. Owing to these minor differences in chemical structure, the four molecules can be expected to have relatively similar stabilities. Stability data from independent studies or pharmaceutical laboratories support this ([16–18]). The majority of studies report that daunorubicin, doxorubicin and epirubicin remain stable for between 8 and 28 days at concentrations ranging from 0.01 mg/mL to 0.1 mg/mL, when stored at refrigerated or room temperature. The type of container (infusion bags made of polyvinyl chloride (PVC), polyethylene (PE) or polypropylene (PP)) and the nature of the contents (0.9% NaCl or 5% Dex) do not affect stability. All of these studies were carried out protected from light, demonstrating a common photosensitivity amongst these molecules. Accelerated degradation studies carried out to establish the stability-indicating capacity of chromatographic methods also highlight a similar sensitivity of doxorubicin ([19]) and daunorubicin ([20]) to alkaline, acidic and oxidative conditions.

As a group of experts has described ([6,15]), the shelf-life should be validated taking chemical, physical and microbiological data into consideration. Microbiological stability depends on the preparation’s ability to support the growth of microorganisms. As demonstrated by Krämer in 1998 [21], Idarubicin has some antimicrobial activity, but this may be limited against some microorganisms (bactericidal effect at 0.07 mg/mL after 120 hours of incubation on *Staphylococcus Aureus*, *Enterococcus Faecium* and *Candida Albicans* but *Pseudomonas Aeruginosa* remains viable). The risk of persistent microbiological contamination is therefore not zero, and this risk must be evaluated considering the production process used and the qualification results mentioned above. In fact, microbiological stability depends also on the preservation of sterility in the final container which much be evaluated by microbiological and physical integrity tests ([22]). Finally, the initial sterility of the preparation may involve the application of Good Manufacturing Practices and are very closely linked to the practices of centralized units: production process, ISO 5 equipment used, closed systems used, adding of a clamped infusion set to the bags or not, operator training and validation, disinfection procedure, secondary packaging used, storage conditions etc… For these reasons, it is recommended to conduct a risk assessment of the preparation and storage process; to regularly validate the preparation process (process simulation tests with culture media), the staff members (conducting media fill tests) and the equipment (microbiological performance qualification) [21].

## Conclusion

The present study provides robust data on the stability of idarubicin preparations administered in bags for hematology indications, and complies with current requirements in terms of stability studies. It updates the data available to date and demonstrates that idarubicin preparations are chemically and physically stable for up to 30 days at low (0.04 mg/mL) and high (0.2 mg/mL) concentrations in 0.9% NaCl and 5% Dex polyolefin-type bags (Freeflex + ®) for infusion, protected from UV, with no effect of temperature during storage (refrigeration 5 ± 3 °C and room temperature 22 ± 3 °C). This will enable hospital pharmacies to prepare medicines safely, optimise advanced preparation processes, and will also provide data in the event of non-compliance with storage conditions.

## Supporting information

S1 Fig Chemical structure of idarubicin hydrochloride. (TIF)

S2 Fig Heating degradation. (TIF)

S3 Fig Oxidative degradation. (TIF)

S4 Fig Acidic degradation. (TIF)

S5 Fig Alkaline degradation. (TIF)

S6 Fig Photodegradation. (TIF)

S7 Fig Physicochemical stability study results at 0.04 mg/mL (A) and 0.2 mg/mL (B). (TIF)

S8 Fig Production of idarubicine for pediatric and adult onco-hematology patients in our University Hospital between 2016–2023. (TIF)

S1 Table pH measurement over time. (DOCX)

S2 Table Osmolality measurement over time. Results expressed in mOsm/kg; Av  =  Average; S.D.  =  Standard deviation; p-value (Mann-Whitney test, significance of 0.05).
(DOCX)

S3 Table Determination of the number of sub-visible particles. Results expressed in number of particles; Av = Average; S.D. = Standard deviation.
(DOCX)

S1 Data Study data. (XLSX)
