# Supplementary material for: One-month physicochemical stability study of idarubicin prepared in polyolefin-type bags for the treatment of hematological diseases
Source: PLoS One. 2026 Aug 28;21(8):e0356906. doi: 10.1371/journal.pone.0356906 (PMC13524237; doi:10.1371/journal.pone.0356906)
Supplement: Supplementary file 1 [file pone.0356906.s009.docx]

**S1 Table: pH measurement over time**

|  | | | DO | | D15 | | D30 | |
| --- | --- | --- | --- | --- | --- | --- | --- | --- |
| Diluent | Concentration | Preservation | Av | S.D. | Av | S.D. | Av | S.D. |
| NaCl  0.9 % | 0.04 mg/mL | 5 ± 3 °C | 5.70 | 0.04 | 5.61 | 0.13 | 5.90 | 0.12 |
|  |  | 22 ± 3 °C | 5.50 | 0.23 | 5.00 | 0.08 | 5.49 | 0.02 |
|  | 0.2 mg/mL | 5 ± 3 °C | 4.60 | 0.01 | 4.46 | 0.03 | 4.67 | 0.12 |
|  |  | 22 ± 3 °C | 4.63 | 0.07 | 4.56 | 0.00 | 4.57 | 0.03 |
| Dextrose 5 % | 0.04 mg/mL | 5 ± 3 °C | 4.42 | 0.01 | 4.47 | 0.04 | 4.39 | 0.08 |
|  |  | 22 ± 3 °C | 4.41 | 0.04 | 4.48 | 0.07 | 4.32 | 0.04 |
|  | 0.2 mg/mL | 5 ± 3 °C | 4.17 | 0.03 | 4.26 | 0.14 | 4.15 | 0.01 |
|  |  | 22 ± 3 °C | 4.18 | 0.04 | 4.20 | 0.04 | 4.17 | 0.01 |

Results expressed in pH units;	Av = Average S.D. = Standard deviation
