# Supplementary material for: One-month physicochemical stability study of idarubicin prepared in polyolefin-type bags for the treatment of hematological diseases
Source: PLoS One. 2026 Aug 28;21(8):e0356906. doi: 10.1371/journal.pone.0356906 (PMC13524237; doi:10.1371/journal.pone.0356906)
Supplement: Supplementary file 2 [file pone.0356906.s010.docx]

**S2 Table: osmolality measurement over time**

|  | | | DO | | D15 | | D30 | |
| --- | --- | --- | --- | --- | --- | --- | --- | --- |
| Diluent | Concentration | Temperature | Av | S.D. | Av | S.D. | Av | S.D. |
| NaCl  0.9 % | 0.04 mg/mL | 5 ± 3 °C | 282.3 | 0.6 | 279.0 | 1.0 | 283.0 | 1.0 |
|  |  | 22 ± 3 °C | 282.0 | 0.0 | 279.0 | 0.0 | 283.0 | 1.0 |
|  | 0.2 mg/mL | 5 ± 3 °C | 283.0 | 0.0 | 284.0 | 1.0 | 285.3 | 4.2 |
|  |  | 22 ± 3 °C | 282.3 | 0.6 | 283.0 | 1.2 | 283.0 | 0.6 |
| Dextrose 5 % | 0.04 mg/mL | 5 ± 3 °C | 294.7 | 0.6 | 291.3 | 1.5 | 294.3 | 1.2 |
|  |  | 22 ± 3 °C | 294.0 | 1.0 | 291.0 | 1.7 | 294.0 | 0.0 |
|  | 0.2 mg/mL | 5 ± 3 °C | 293.7 | 1.8 | 292.7 | 3.0 | 289.7 | 0.6 |
|  |  | 22 ± 3 °C | 292.0 | 0.0 | 292.3 | 0.6 | 292.0 | 1.7 |

Results expressed in mOsm/kg;	Av = Average; S.D. = Standard deviation
