# Supplementary material for: One-month physicochemical stability study of idarubicin prepared in polyolefin-type bags for the treatment of hematological diseases
Source: PLoS One. 2026 Aug 28;21(8):e0356906. doi: 10.1371/journal.pone.0356906 (PMC13524237; doi:10.1371/journal.pone.0356906)
Supplement: Supplementary file 3 [file pone.0356906.s011.docx]

**S3 Table: determination of the number of sub-visible particles**

| Conditions | | | 0 | | | | 15 | | | | 30 | | | |
| --- | --- | --- | --- | --- | --- | --- | --- | --- | --- | --- | --- | --- | --- | --- |
| Diluent | Concentration | Temperature | ≥10 µm | | ≥25 µm | | ≥10 µm | | ≥25 µm | | ≥10 µm | | ≥25 µm | |
|  |  |  | Av | S.D. | Av | S.D. | Av | S.D. | Av | S.D. | Av | S.D. | Av | S.D. |
| NaCl 0.9 % | 0.04 mg/mL | 5 ± 3 °C | 814.45 | 256.85 | 26.67 | 14.81 | 855.56 | 133.90 | 24.45 | 11.10 | 909.44 | 218.47 | 21.11 | 16.44 |
|  |  | 22 ± 3 °C | 840.00 | 271.67 | 64.44 | 45.78 | 832.22 | 127.61 | 26.11 | 21.50 | 735.00 | 288.01 | 53.89 | 62.63 |
|  | 0.2 mg/mL | 5 ± 3 °C | 1510.00 | 1356.20 | 22.78 | 8.22 | 525.55 | 186.32 | 11.11 | 6.73 | 827.78 | 627.19 | 51.11 | 52.98 |
|  |  | 22 ± 3 °C | 1085.56 | 239.68 | 75.00 | 64.96 | 1016.67 | 237.93 | 27.22 | 12.51 | 751.11 | 171.94 | 24.44 | 7.88 |
| Dextrose 5 % | 0.04 mg/mL | 5 ± 3 °C | 1510.00 | 1356.20 | 22.78 | 8.22 | 525.55 | 186.32 | 11.11 | 6.73 | 827.78 | 627.19 | 51.11 | 52.98 |
|  |  | 22 ± 3 °C | 1085.56 | 239.68 | 75.00 | 64.96 | 1016.67 | 237.93 | 27.22 | 12.51 | 751.11 | 171.94 | 24.44 | 7.88 |
|  | 0.2 mg/mL | 5 ± 3 °C | 3044.11 | 955.54 | 11.11 | 16.44 | 986.67 | 128.33 | 10.00 | 1.67 | 973.89 | 265.17 | 4.44 | 0.96 |
|  |  | 22 ± 3 °C | 2067.78 | 737.20 | 15.00 | 3.33 | 1131.67 | 418.58 | 12.22 | 10.58 | 790.00 | 161.58 | 28.34 | 7.64 |

Results expressed in number of particles; Av = Average; S.D. = Standard deviation
