# Supplementary material for: One-month physicochemical stability study of idarubicin prepared in polyolefin-type bags for the treatment of hematological diseases
Source: PLoS One. 2026 Aug 28;21(8):e0356906. doi: 10.1371/journal.pone.0356906 (PMC13524237; doi:10.1371/journal.pone.0356906)
Supplement: Supplementary file 4 [file pone.0356906.s013.docx]

Dear editor,

Thank you for asking me to review the manuscript “One-month physicochemical stability study of idarubicin prepared in polyolefin bags for the treatment of hematological diseases.”

This work involves determining the stability of idarubicin bag preparations. The data available to date are outdated and poorly documented. Stability data make it possible to plan preparations in advance and improve the use of these drugs in healthcare settings.

I find that the study does not analyze, or analyzes only to a limited extent, the factors causing degradation, particularly in the discussion section, which does not adequately describe the chemical phenomena that may occur in terms of molecule degradation.

Given the results of the forced degradation study, the data obtained are not very informative about the measures to be taken and the conditions to be avoided in order to prevent degradation of the molecule.

In the absence of data, it would also have been interesting to explore the stability of drugs with similar chemical structures in order to potentially predict the mechanisms of degradation of the molecule. Compared to other anthracyclines, do we see the same degradation profiles? And with the same effect depending on the material, concentration, and solvent?

I understand that there was no degradation of any kind during the stability study.

Regarding the chromatogram results: were there spectra with different wavelengths with the DAD? Because it is surprising that no degradation was found.

If no signs of degradation were found, why not conduct a longer stability study in order to characterize any potential degradation (and, moreover, store the product for longer)?

Furthermore, the term “polyolefin” is likely to mislead the reader, considering that all types of polyolefin were tested in contact with the molecule of interest, which is not what was done: only one free flex + model was tested, with polypropylene in contact with the molecule of interest. Why not test other materials (PVC, EVA, etc.)?

Finally, as a general comment, I did not find in the manuscript whether a specific protocol was implemented to protect the operators who carried out the control and stability study (handling of samples, risk of environmental contamination, etc.).

Minor corrections:

In the title: is it really necessary to specify “for the treatment of hematological diseases”?

P1 Not all authors have included their affiliation.

In Figure 2: how can we explain the decrease in concentration on Day 15 at a concentration of 0.04 mg/mL at 20°C, which we do not see subsequently?

L 57: provide a little more detail on the findings of the Beijnen stability study. The Handbook of Injectable Drugs also identifies another stability study not referenced in your manuscript: Farmitalia Carlo Erba. Stability of 4 demethoxydaunorubicin hydrochloride reconstituted solutions with water for injections, sodium chloride, dextrose. Milan, Italy 1986. It might be interesting to analyze and include this work.

L 73: Does idarubicin not exist in standard SCR quality (EDQM pharmacopoeia)?

L19: There is a generic drug in France. What are the differences, and can the data obtained be extrapolated between the different forms available?

L106: Beyond purity, is there water in the powder?

L106: The description of the Milli-Q water production system should be detailed earlier, for example at the production of mobile phases, as we imagine that this water was used in the preparation of phosphate buffer solutions.

L110: “a serie” and not “a series”

L116: Were the QC points achieved with API powder from Sigma Aldrich?

Is there a monograph in the pharmacopoeia for idarubicin?

L140: Define the parameters that justify satisfactory separation at the peak level.

It is unusual to group the acceptance criteria in the same paragraph; I am surprised by the detection of degradation products as a criterion for non-acceptance; For pH and osmolarity, there could be a statistically significant difference without any repercussions, depending also on the number of samples and the power; it may therefore be more appropriate to set threshold values as specifications; for the pH specification, it is important to adapt it to the presence or absence of buffers in the preparation.

For method optimization, it may be useful to determine the resolution coefficients;

Zoom on s2 poorly represented

Deg 2 on s3: unresolved peak

Deg 3 and 4 S5: unresolved peaks

S6: the results show unresolved peaks and the retention time varied greatly; is there an explanation for this? Why not shorten the 24-hour time considerably in the hope that the degradation would not be too significant?

For representation of stability over time: the points in time on the x-axis should be spaced appropriately.

L191: the significant number of LOD and LOQ is too high and inappropriate.

L261: not “maintained” because n is not a pH buffer.

L 263: Why is polyolefin the preferred format for chemotherapy bags?

There is no bibliographic reference to support this assertion.

L 270: The paragraph on osmolarity is not useful and contributes little to the discussion itself; in fact, the starting specialty is isotonic and placed in a larger isotonic volume, so it is not surprising to have an isotonic solution in the end.

L277 The particle values are very high at D0, particularly for glucose, and decrease thereafter. Is there an explanation or a causal reason for this phenomenon?

You indicate that this may be related to the control environment not being sterile; however, did this change during subsequent measurements? This is potentially related to an initial release of particles from the material, but this has not been discussed; did you test several batches of bags?

L280 What was the assay method used in this study and was it stability indicating?

L 283 I find the method for determining the concentrations tested useful with the explanation; I think it could be included in the introduction or materials and methods section?
