# Supplementary material for: One-month physicochemical stability study of idarubicin prepared in polyolefin-type bags for the treatment of hematological diseases
Source: PLoS One. 2026 Aug 28;21(8):e0356906. doi: 10.1371/journal.pone.0356906 (PMC13524237; doi:10.1371/journal.pone.0356906)
Supplement: Supplementary file 5 [file pone.0356906.s014.docx]

The study is well conducted and results are very usefull for hospital pharmacists

I recommend the publication of the article after a minor review.

Kindly find below my comments and recommandation

Line 70 : bags were… It would be better to formulate as : For solution dilution, Freeflex bags were purchased from … The infusion bags were made of…..

Line 80-82: it can be clearer for the reader if short sentences are used

Line 118: same instead of sale

Line 202: a table with results and the mass balance for each condition should be added and table 1 should give more information

Line 207; 209; 2016: degradation products should be named differently for exemple DEG1 and DEG2 for the S2 fig then DEG3 and DEG4 for the S3fig … as they are not the same.

Are degradation products described in the literature or in pharmacopoeia?

Line 231: is the concentration of the sample enough to detect degradation product? We usually use more concentrated solution for that. There is no information about the sample solution. Are solutions analyzed without any dilution? And at this concentration, has the limit of detection of degradation products been tested? What are the specifications for degradation products? Such information should be given for stability indicating method. The limits have been specified only for the assay for the active substance

Line 239: and for the dextrose bags?
