# Supplementary material for: One-month physicochemical stability study of idarubicin prepared in polyolefin-type bags for the treatment of hematological diseases
Source: PLoS One. 2026 Aug 28;21(8):e0356906. doi: 10.1371/journal.pone.0356906 (PMC13524237; doi:10.1371/journal.pone.0356906)
Supplement: Supplementary file 6 [file pone.0356906.s015.docx]

**Point by point response to editor and reviewers**

Dear editor, dear reviewers,

On behalf of all the authors, we would first like to thank you very much for the time you have spent reviewing, for providing feedback on our work and for your encouraging comments. All of your comments have been carefully considered, we have made additional experiment and conducted further literature reviews.

This document includes all of your comments and our responses to them. To make it easier to review: our answers are written in blue; additions to the manuscript are written in italics; we have specified the lines where changes have been made. The line numbers correspond to the marked-up copy of our manuscript.

We hope that the answers provided will meet your expectations and the revised manuscript will now be considered appropriate for publication in PLOS ONE.

We are available to provide any further information you may need

Best regards

Dr. Guillaume Bouguéon

Corresponding Author

Table of contents

[Email from the academic editor-in-chief	2](#_Toc235255029)

[Additional Editor Comments:	3](#_Toc235255030)

[Response to reviewer 1:	9](#_Toc235255031)

[Response to reviewer 2:	27](#_Toc235255032)

[Response to Reviewer 3:	30](#_Toc235255033)

[Request for an extension of the review period	36](#_Toc235255034)

# Email from the academic editor-in-chief

PONE-D-26-07865

One-month physicochemical stability study of idarubicin prepared in polyolefin bags for the treatment of hematological diseases.

PLOS One

Dear Dr. Bouguéon, 

Thank you for submitting your manuscript to PLOS ONE. After careful consideration, we feel that it has merit but does not fully meet PLOS ONE’s publication criteria as it currently stands. Therefore, we invite you to submit a revised version of the manuscript that addresses the points raised during the review process.

Please submit your revised manuscript by Apr 26 2026 11:59PM. If you will need more time than this to complete your revisions, please reply to this message or contact the journal office at [plosone@plos.org](mailto:plosone@plos.org). When you're ready to submit your revision, log on to <https://www.editorialmanager.com/pone/> and select the 'Submissions Needing Revision' folder to locate your manuscript file.

Please include the following items when submitting your revised manuscript:

- A letter that responds to each point raised by the academic editor and reviewer(s). You should upload this letter as a separate file labeled 'Response to Reviewers'.
- A marked-up copy of your manuscript that highlights changes made to the original version. You should upload this as a separate file labeled 'Revised Manuscript with Track Changes'.
- An unmarked version of your revised paper without tracked changes. You should upload this as a separate file labeled 'Manuscript'.

If you would like to make changes to your financial disclosure, please include your updated statement in your cover letter. Guidelines for resubmitting your figure files are available below the reviewer comments at the end of this letter.

If applicable, we recommend that you deposit your laboratory protocols in protocols.io to enhance the reproducibility of your results. Protocols.io assigns your protocol its own identifier (DOI) so that it can be cited independently in the future. For instructions see: [https://journals.plos.org/plosone/s/submission-guidelines#loc-laboratory-protocols](https://track.editorialmanager.com/CL0/https:%2F%2Fjournals.plos.org%2Fplosone%2Fs%2Fsubmission-guidelines%23loc-laboratory-protocols/1/010f019ce0fae0c6-6c377dba-a4b9-4182-abda-0fc7ce7c7f74-000000/Q5Y1NQnarvy47Lo8znEScR4jg4zI45eSTNHNPhc7sxY=254). Additionally, PLOS ONE offers an option for publishing peer-reviewed Lab Protocol articles, which describe protocols hosted on protocols.io. Read more information on sharing protocols at [https://plos.org/protocols?utm_medium=editorial-email&utm_source=authorletters&utm_campaign=protocols](https://track.editorialmanager.com/CL0/https:%2F%2Fplos.org%2Fprotocols%3Futm_medium=editorial-email%26utm_source=authorletters%26utm_campaign=protocols/1/010f019ce0fae0c6-6c377dba-a4b9-4182-abda-0fc7ce7c7f74-000000/czjQ7Th-LRvz74qEzj3lLDnUb4hVAhgkmhnhG_4O5Fo=254).

We look forward to receiving your revised manuscript.

Kind regards,

Additional Editor Comments:

Dear Authors,

According to reviewers reports, I recommend you (in addition to your point-to point answer to reviewers comments) to improve the forced degradation experiments part as reported by all reviewers. Reviewer 1 have identified several flaws which probably require additional experiments.

From my point of view, and after reading this part:

For light and alkaline degradation experiments, idarubicine was too much degraded. Generally, during forced degradation experiments, a degradation approximately equal to 10-30% is targeted to identify primary degradation products and to obtain a well-resolved mixture. Light and alkaline experiments should be probably done again under new conditions.

For oxidative experiment, an unresolved peak is observed (DEG 2, RT 2.707). It appears as a limitation of your work unless new data are provided. Moreover, two peaks were reported to be related to H2O2 (2.707 and 4.300)? Are you sure? H2O2 in excess is not the first big peak with RT 2.0 min?

For light experiment (S6), why is there a "NaOH" peak at 1.873 min?

Improve degradation products numbering as requested by reviewers 2 and 3 (don't reuse number).

Moreover, I recommend you to provide RRT rather than retention time, and you could also identify degradation products which appear under several degradation conditions.

In accordance with your recommendations and in response to the reviewers' questions, we were able to conduct further investigations to refine the evaluation of our assay method as a stability indicating method. Additional experiments were carried out under oxidative, alkaline and light conditions. For each condition, several variations in the degradation parameters were tested. We are presenting the results that we believe best meet your expectations.

Below is an **updated summary table** of the forced degradation results. At the request of Reviewer 2, the table has been added to the main text and numbered “Table 2”.

| Type of degradation | Conditions | Percentage of degradation |
| --- | --- | --- |
| Heating stress | 80 °C | 38.2 % 4 h |
| Oxidative | H_2_0_2_ 7.5 % | 22.7 % - 30min |
| Acidic | HCl 0.1 M – 80 °C | 34.3 % - 5 min |
| Alkaline | NaOH 1 M – 70 °C | 15.2 % - 1 min |
| Photolytic stress | UV – 20 °C | 19.3 % - 2h |

For comparison, here are **the initial results** we shared to you

| Type of degradation | Conditions | Percentage of degradation |
| --- | --- | --- |
| Heating stress | 80 °C | 38.2 % 4 h |
| Oxidative | H_2_0_2_ 30 % | 30.0 % - 4 h |
| Acidic | HCl 0.1 M – 80 °C | 34.3 % - 5 min |
| Alkaline | NaOH 1 M – 80 °C | 73.8 % - 2 min |
| Photolytic stress | UV – 20 °C | 99.7 % - 24 h |

As you can see, we have adjusted the degradation conditions to achieve lower degradation rates. Note that, since the conditions have changed somewhat, we did not obtain exactly the same degradation products at the same retention times as in our previous submission.

The chromatograms are provided below in the additional experiment parts dedicated to each study condition.

**Degradation products naming**

The naming of degradation products was revised as follow to make them easier for readers to understand:

- “Deg H + number” for heating conditions
- “Deg Ox + number” for oxidative conditions
- “Deg Ac + number” for acidic conditions
- “Deg Alk + number” for alkaline conditions
- “Deg L + number” for light conditions

**Relative retention time (RRT)**

You advised us to use relative retention time (RRT) instead of retention time (RT) when evaluating the stability of our assay method.

This parameter helps to account for any fluctuations in RT that may be inherent to the assay method and the chromatography instrument used.

The calculation of RRT relies on the use of an internal standard. It is obtained by dividing the RT of an internal standard by the RT of the peak under study.

For example, using RRT could allow us to confirm that a degradation peak observed at time X during one analysis is the same as the one observed during another analysis under the same conditions but at a slightly different retention time Y.

In the context of our study, the objective is to degrade the molecule under five different conditions and to verify that the molecule of interest is indeed separated from the degradation products. The procedures performed and presented here yielded these results. For each condition, the resulting chromatogram was visually compared to the chromatogram of an idarubicin reference solution, prepared on the same day. In addition, the “peak purity match” was determined to verify the presence of a single molecule at a given retention time.

To the best of our knowledge, using RTT to assess the stability-indicating capabilities of our assay method would not provide us with any more information than we have already obtained.

Of course, in the context of conducting the stability study, if several degradation products had been observed and we had wanted to track them accurately over time, we would have used RTT to strengthen our analysis.

**Additional experiment for oxidative conditions:**

Editor’s comments: For oxidative experiment, an unresolved peak is observed (DEG 2, RT 2.707). It appears as a limitation of your work unless new data are provided. Moreover, two peaks were reported to be related to H2O2 (2.707 and 4.300)? Are you sure? H2O2 in excess is not the first big peak with RT 2.0 min?

The new experiments showed that degradation under oxidative conditions was rapid with 30% H₂O₂ (nearly 50% after 1 hour). That is why we repeated these tests using H₂O₂ diluted to one-quarter strength, at 7.5%. These tests showed a degradation of 22.7% after 30 minutes. These are the results we are presenting to you with a clearer separation between H₂O₂ /Idarubicine and degradation product. We can also confirm that H₂O₂ exhibits several peaks, as you can see in the analysis of a sample containing 7.5% H₂O₂ under the same condition.

**First draft submitted**

**New draft submitted**

H_2_0_2_ 7.5% analysis alone (not submitted)

**Additional experiment for alkaline conditions:**

Editor’s comments: For light and alkaline degradation experiments, idarubicine was too much degraded. Generally, during forced degradation experiments, a degradation approximately equal to 10-30% is targeted to identify primary degradation products and to obtain a well-resolved mixture. Light and alkaline experiments should be probably done again under new conditions.

For this experiment, we reduced the temperature to 70°C and the time to 1 minute. As a result, the degradation rate dropped from 73.8% to 15.2%.

Despite our various experiments, the Deg Alk1 degradation product remains close to the idarubicin peak. We were unable to completely separate the two peaks without changing the chromatographic conditions.

**First draft submitted**

**New draft submitted**

**Additional experiment for light conditions:**

Editor’s comments: For light and alkaline degradation experiments, idarubicine was too much degraded. Generally, during forced degradation experiments, a degradation approximately equal to 10-30% is targeted to identify primary degradation products and to obtain a well-resolved mixture. Light and alkaline experiments should be probably done again under new conditions.

For this experiment, we reduced the exposure time to 2 hours. We observed a 19.3% reduction in the peak and began to see degradation products appear both upstream and downstream of the reference peak.

Some degradation products could not be distinguished from one another; however, they are clearly distinguishable from the idarubicin peak.

**First draft submitted**

**New draft submitted**

Editor’s comments: For light experiment (S6), why is there a "NaOH" peak at 1.873 min?

This is an error related to the Chromeleon software that occurred following the sequential analysis of samples under alkaline conditions and exposure to light conditions. The error has been corrected and does not appear in the new version of the figure.

In summary, by modifying the degradation conditions, we were able to reduce the degradation rate of idarubicin to acceptable levels.

We are aware that some degradation products have not been separated from one another using our current technique, as you can see in some of the chromatograms.

We did not want to change parameters related to the composition of the mobile phase or the temperature, for example, as this would have completely undermined the entire chemical stability study already conducted.

Nevertheless, the initial objective was to determine whether our technique was capable of separating idarubicin from its degradation products. This is what we achieved.

An additional factor supporting our findings is our instrument’s ability to determine peak purity match. This factor allows us to determine whether a peak consists of one or more molecules. In all of our experiments (method development, determination of stability indicator capacity, stability study), the purity of the idarubicin peaks was greater than 99.5%.

To take this further, we would need to work with a more powerful detector, such as a mass spectrometer. One of the initial objectives was to use an affordable device so that this work and our method could be replicated by as many hospital facilities as possible.

We hope these results meet your expectations. We have therefore made the necessary changes to the Materials and Methods (part “Forced degradation study” between line 169 to 189) and Results (part “Stability-indicating method” between line 260 to 288) sections and to the figures (S2 to S6 figures).

# Response to reviewer 1:

Dear editor,

Thank you for asking me to review the manuscript “One-month physicochemical stability study of idarubicin prepared in polyolefin bags for the treatment of hematological diseases.”

This work involves determining the stability of idarubicin bag preparations. The data available to date are outdated and poorly documented. Stability data make it possible to plan preparations in advance and improve the use of these drugs in healthcare settings.

I find that the study does not analyze, or analyzes only to a limited extent, the factors causing degradation, particularly in the discussion section, which does not adequately describe the chemical phenomena that may occur in terms of molecule degradation.

Given the results of the forced degradation study, the data obtained are not very informative about the measures to be taken and the conditions to be avoided in order to prevent degradation of the molecule.

We would like to thank the reviewer for this comment and will endeavour to provide readers with further clarification.

The following clarifications have been added to the manuscript (between line 386 to 417):

*“In the present study, all factors that may influence the physical and chemical stability of injectable preparations (e.g. temperature, hydrolysis, redox reactions, photosensitivity, precipitation) have been tested (see ref 1 below).*

*The results of the accelerated degradation study show that the molecule is sensitive to extreme temperatures (80°C). Our study was not conducted at temperatures exceeding 25°C. We therefore recommend that transport and storage take place at controlled temperatures. Nevertheless, given the short duration of administration recommended by the suppliers (approximately ten minutes) (see ref 2 below), a temperature deviation of <35°C within this time frame should not significantly affect the stability of the molecule.*

*Idarubicin is sensitive to acidic (0.1N HCl) and extremely basic (1M NaOH) conditions. The various hydroxyl, amine and ketone groups, as well as the glycosidic bond and the aromatic ring, may therefore undergo significant chemical reactions under these conditions, leading to the formation of degradation products. The pH monitoring showed an acceptable variation around the values measured at Day 0 and did not reveal any significant formation of H^+^ or OH^-^ ions over the study period. Furthermore, as no degradation products were observed within the detection limits of DAD spectroscopy, idarubicin was therefore not subject to significant hydrolysis.*

*When preparing the samples for the study, the residual air in the infusion bags was not removed. The accelerated degradation study demonstrated degradation under oxidative conditions caused by H₂O₂. In light of the results of the stability study, the residual air in the bags did not cause any oxidation or affect the stability of idarubicin. This air therefore does not need to be removed when preparing the infusion bags.*

*With regard to light sensitivity, the accelerated degradation study demonstrated the instability of the idarubicin molecule when exposed to UV radiation. For this reason, the study was conducted by protecting the infusion bags with light-protective packaging. We therefore recommend that users protect their bags. If the administration is brief, as recommended by the suppliers, the idarubicin will pass through an unprotected infusion tube quickly and is very likely to undergo minimal degradation. Should the administration take longer than 30 minutes for various reasons, we recommend using a light-protective infusion tube.*

*Finally, during our study, we did not observe any precipitates either with the naked eye or under the microscope. Similarly, no change in colour was observed. Nevertheless, should any change in colour or the appearance of a precipitate be observed under clinical conditions, administration of the product should be discontinued and further investigations carried out to determine the causes. “*

References:

- Ref 1: SFPC/GERPAC. Methodological guidelines for stability studies of hospital pharmaceutical preparations, Part I : Liquid preparations. [Internet]. 2013. Available from: <http://www.gerpac.eu/IMG/pdf/%0Aguide_stabilite_anglais.pdf>
- Ref 2: ANSM. Zavedos résumé des caractéristiques du produit [Internet]. 2024 [cited 2024 Dec 19]. Available from: <http://agence-prd.ansm.sante.fr/php/ecodex/frames.php?specid=65133315&typedoc=R&ref=R0321035.htm>

In the absence of data, it would also have been interesting to explore the stability of drugs with similar chemical structures in order to potentially predict the mechanisms of degradation of the molecule. Compared to other anthracyclines, do we see the same degradation profiles? And with the same effect depending on the material, concentration, and solvent?

The following clarifications have been added to the manuscript (between line 419 to 433):

*“Idarubicin is an anthracycline; the main representatives of this class used in oncology are doxorubicin, epirubicin and daunorubicin. Idarubicin is an analogue of daunorubicin, differing in the absence of a methoxy group at the 4’ position. Doxorubicin differs from daunorubicin in that it has an additional hydroxyl group at the 14’ position. Epirubicin, meanwhile, is an epimer of doxorubicin at the C4’ position of the sugar. Owing to these minor differences in chemical structure, the four molecules can be expected to have relatively similar stabilities.*

*Stability data from independent studies or pharmaceutical laboratories support this (Ref 1 to 3). The majority of studies report that daunorubicin, doxorubicin and epirubicin remain stable for between 8 and 28 days at concentrations ranging from 0.01 mg/mL to 0.1 mg/mL, when stored at refrigerated or room temperature. The type of container (infusion bags made of polyvinyl chloride (PVC), polyethylene (PE) or polypropylene (PP)) and the nature of the contents (0.9% NaCl or 5% Dex) do not affect stability.*

*All of these studies were carried out protected from light, demonstrating a common photosensitivity amongst these molecules. Accelerated degradation studies carried out to establish the stability-indicating capacity of chromatographic methods also highlight a similar sensitivity of doxorubicin (ref 4) and daunorubicin (ref 5) to alkaline, acidic and oxidative conditions.”*

References:

- Ref 1: Doxorubicin monography from Stabilis 4.0. https://www.stabilis.org/Monographie.php?IdMolecule=73
- Ref 2: Daunorubicin monography from Stabilis 4.0. https://www.stabilis.org/Monographie.php?IdMolecule=57
- Ref 3: Epirubicin monography from Stabilis 4.0. https://www.stabilis.org/Monographie.php?IdMolecule=75
- Ref 4: Doxorubicin Stability‑indicating Method and its Main Degradation Products *In vitro* Toxicity Mariah de Almeida Ultramari1 · Ariane Rivellis Julio1 · Larissa Souza Passos2 · Alexander Ossanes de Souza2 · Núbia Pereira da Silva2 · Paloma Nathane Nunes de Freitas2 · Ernani Pinto1,2,3
- Ref 5: A stability-indicating, ion-pairing, reversed-phase liquid chromatographymethod for studies of daunorubicin degradation in i.v. infusion fluidsR. Respauda,b,c,∗, L. Quenuma,c,1, C. Plichonb,2, J.F. Tournamillea,b,c,2, E. Gyanb,c,d,3, D. Antierb,2,M.C. Viaud-Massuarda

Journal of Pharmaceutical and Biomedical Analysis 83 (2013) 164– 170

I understand that there was no degradation of any kind during the stability study.

Regarding the chromatogram results: were there spectra with different wavelengths with the DAD? Because it is surprising that no degradation was found.

The DAD detector used enabled the analysis of each sample over a wavelength range between 190 and 400 nm. The chromatograms presented represent the analyses performed at 254 nm, the optimal wavelength for the analysis of idarubicin.

For each observed peak, the software compares all spectral data obtained across the study wavelength range and determines a “peak purity match.” This factor is expressed as a score out of 1000. This allows for the identification of multiple molecules with different spectra but the same retention time. The closer the score is to 1000, the purer the peak. A score deviating from 1000 indicates the presence of multiple molecules eluting at the same retention time.

During our stability study, no degradation products were observed on the chromatograms, and the peak purity factor was greater than 999.5‰ (or 99.5%), demonstrating the absence of co-elution between idarubicin and a degradation product.

If no signs of degradation were found, why not conduct a longer stability study in order to characterize any potential degradation (and, moreover, store the product for longer)?

Thank you for your insightful comment.

It is true that, given the results obtained, we could have continued the study. However, the study was originally planned to last 30 days, and we did not have enough samples at the time to repeat all the tests that had been conducted.

To echo the arguments put forward by our colleagues at the Angers University Hospital Center (see Bourcier *et al.* 2022), 30 days is a period that seems long to us in a hospital setting, as it allows for drug preparation and facilitates pharmacy organization, including transport services to hospital wards.

We understand that information on stability beyond 30 days could be useful for certain facilities. That is why we are taking your comment into account for future work.

References:

Bourcier B, Lagarce F, Lebreton V. Stability of a high-concentrated aqueous solution of idarubicin stored in a polypropylene syringe for transarterial chemoembolization. J Pharm Biomed Anal. 2022;210:114543. doi:10.1016/j.jpba.2021.114543 PubMed PMID: 34991044.

Furthermore, the term “polyolefin” is likely to mislead the reader, considering that all types of polyolefin were tested in contact with the molecule of interest, which is not what was done: only one free flex + model was tested, with polypropylene in contact with the molecule of interest.

The term “polyolefin” refers to a family of thermoplastic polymers produced by the polymerization of olefins (unsaturated hydrocarbons, such as ethylene or propylene). Bags made from polyolefins therefore contain polypropylene or polyethylene, such as the Freeflex+® (primary internal film: polypropylene; secondary external film: polypropylene/polyethylene admixture).

It is true that we have not conducted studies on several types of polyolefin bags. Other examples that could be mentioned include Viaflo, Macopharma and Easyflex, for instance.

Stabilis, the leading website on stability studies, cites numerous studies that have highlighted this generic term, “polyolefin.”

- <https://www.stabilis.org/Recherche.php?rechercheGlobale=polyol%C3%A9fin&x=0&y=0>

We can therefore cite examples of published stability studies that have been conducted on polyolefins IV bags.

- <https://www.degruyterbrill.com/document/doi/10.1515/pthp-2022-0007/html>
- <https://journals.sagepub.com/doi/10.1177/10781552231152618?url_ver=Z39.88-2003&rfr_id=ori:rid:crossref.org&rfr_dat=cr_pub%20%200pubmed>
- <https://journals.lww.com/ejop/Fulltext/2022/06000/Physicochemical_stability_of_pemetrexed_diarginine.1.aspx>
- <https://gabi-journal.net/extended-stability-of-the-trastuzumab-biosimilar-abp-980-kanjinti-in-polyolefin-bags-and-elastomeric-devices.html>

For all of these studies, a single brand of polyolefin bags was used.

While the term “polyolefin” is fairly generic, we believe that the range of materials used in EVA- or PVC-type bags can vary just as much as it does among suppliers of polyolefin-type bags.

Nevertheless, we understand your point and have:

- adjust the title of our work: “*One-month physicochemical stability study of idarubicin prepared in* ***polyolefin-type bags*** *for the treatment of hematological diseases* ».
- where necessary, we have clarified the term ‘polyolefin’ as follows: “*polyolefin-type bags (Freeflex+®)”.*

Why not test other materials (PVC, EVA, etc.)?

We would like to provide a joint response to this question—another one you raised—and to the comment from Reviewer 3, which echoes your concerns.

**Reviewer 1:** L 263: Why is polyolefin the preferred format for chemotherapy bags? There is no bibliographic reference to support this assertion.

**Reviewer 3:** Page 3, line 64: Why not use PVC bags anyway? They are the most commonly used for all treatments in North America. But you explain later that this isn't the case for chemotherapy in Europe…

Our hospital uses polyolefin bags for the preparation of cytotoxic and non-cytotoxic drugs by the central pharmacy, primarily the Freeflex+® series.

Several hospitals, with which we share the same drug purchasing contracts, also use this same reference. Other alternatives to the Freeflex+ series are also listed in our contracts (e.g., Viaflo®, Easyflex®), but these are also polyolefin bags. We therefore do not use PVC or EVA bags and do not performed study in these bags.

To the best of our knowledge, PVC bags have a bad reputation due to several factors:

- The release of plasticizer :

<https://www.sciencedirect.com/science/article/pii/S0928493117305726>

<https://pmc.ncbi.nlm.nih.gov/articles/PMC6908714/>

<https://ec.europa.eu/health/ph_risk/committees/scmp/documents/out43_en.pdf>

- Drug interactions with these bags

<https://www.sciencedirect.com/science/article/abs/pii/S0731708504005424>

<https://www.sciencedirect.com/science/article/abs/pii/S0378517305000608>

<https://pubmed.ncbi.nlm.nih.gov/33277818/>

- An environmental impact

<https://www.sciencedirect.com/science/article/pii/S0025326X23008263>

Several websites report these points:

- <https://www.vdfmedpack.com/non-pvc-iv-drip-bags-vs-traditional-iv-bags-which-is-safer-for-medical-infusion/>
- <https://www.ciamedical.com/insights/what-material-are-iv-bags-made-of/?srsltid=AfmBOor9zfLoiwNTL1K1Xu3QLlz7sRqgUHpEJ2pRVjGtSi3zIhiYHEam>

Some organizations recommend replacing it with other solutions:

- <https://europe.noharm.org/news/removing-harmful-pvc-healthcare>

It was in this context that we noted that many centers were using polyolefin bags for the preparation of cytotoxic drugs.

We acknowledge, however, that after a more careful review of the literature, we must temper our statements. In fact, we have not found any published studies reporting increased use of these materials. Nor have we found any studies demonstrating the advantages of using polyolefin bags over PVC bags.

That is why we propose to revise our manuscript by removing the sentence that might be confusing to the reader (between line 328 to 330).

Finally, as a general comment, I did not find in the manuscript whether a specific protocol was implemented to protect the operators who carried out the control and stability study (handling of samples, risk of environmental contamination, etc.).

Although this was not specified in our manuscript, collective and individual protective measures were implemented throughout the course of this study. We propose adding the following paragraph to the “Materials and Methods” section of the manuscript (between line 122 to 131).

***“Collective and Individual Protective Measures Against Cytotoxic Risks***

*All staff involved in sample preparation and quality control during the stability study have received training and are authorized to handle cytotoxic medicines.*

*The preparation of cytotoxic drugs was performed in an ISO 5 negative-pressure isolator located within an ISO 7 controlled-atmosphere area. Operators wore appropriate protective clothing for work in these areas: reusable fabric scrubs and gowns, single-use caps, and single-use non-sterile gloves.*

*During the control steps, sample preparation was performed in areas with an unclassified atmosphere. Appropriate collective protective measures were taken: handling under a microbiological safety cabinet class 2, use of sterile drapes. Technicians wore appropriate protective clothing: non-sterile gloves, a single-use lab coat, and a single-use mask.”*

Minor corrections:

In the title: is it really necessary to specify “for the treatment of hematological diseases”?

The primary indications listed in the marketing authorization are in the field of onco-hematology. By specifying this, we sought to distinguish our study from another study conducted using a syringe by the team at Angers University Hospital (see Bourcier *et al.* 2022), which focused on indications for chemoembolization.

Bourcier B, Lagarce F, Lebreton V. Stability of a high-concentrated aqueous solution of idarubicin stored in a polypropylene syringe for transarterial chemoembolization. J Pharm Biomed Anal. 2022;210:114543. doi:10.1016/j.jpba.2021.114543 PubMed PMID: 34991044.

P1 Not all authors have included their affiliation.

We apologize for this error and will check it when the article is resubmitted.

In Figure 2: how can we explain the decrease in concentration on Day 15 at a concentration of 0.04 mg/mL at 20°C, which we do not see subsequently?

In fact, we observed a decrease in pH for the bags prepared at 0.04 mg/mL in NaCl and stored at room temperature on day 15 (5.00; SD 0.08). As a reminder, this result is based on the analysis of 3 independent bags. As can be seen, the standard deviation is low, indicating values that are close to and not widely dispersed around the mean. As shown in Figure 2 and Table S2, this decrease is transient, and the value observed at D0 (5.50; S.D. 0.23) returns to a value nearly identical to that at D30 (5.49, S.D. 0.02). This deviation is surprising because it was not observed under the other study conditions. All samples from the different conditions were analyzed on the same day, under the same conditions, by the same operator and after the instrument had been calibrated. All samples were mixed before analysis and analyzed in less than an hour and a half.

Our preparation does not contain a buffer. 0.9% NaCl solutions have an ionic strength considered to be low to moderate. As a result, it may be sensitive to various factors. We know that pH can be affected by temperature (decrease as the temperature rises), such as the presence of CO_2_ (decrease in pH due to the dissolution of CO_2_). The pH-meter was in a laboratory where the temperature is not monitored. We do not believe this could have influenced the measurements, as we would have observed it in other samples under different conditions.

Some hospital pharmacy teams have observed variations in the pH of hospital-prepared solutions from one batch to the next without any clear explanation, despite further investigation. (See: <https://ejhp.bmj.com/content/ejhpharm/31/Suppl_1/A42.2.full.pdf?utm_source=consensus>).

To date, we have no explanation for this temporary drop in pH. However, this drop is relatively minor and remains within the established range of one pH unit. If this trend had continued, our conclusions would obviously have been different.

L 57: provide a little more detail on the findings of the Beijnen stability study.

We propose expanding the section on the work of Beijnen et al. as follows (between line 58 to 71).

*“Regarding the dilution of idarubicin in infusion bags, the only study available was conducted by Beijnen et al. in 1985. [Beijnen et al. 1985]* *In this article, the stability of idarubicin diluted to a concentration of 0.1 mg/mL in 0.3 % and 0.9 % NaCl, 3.3 % and 5% Dex and Ringer Lactate was assessed. The study was conducted at room temperature and samples were protected from light. Beijnen et al. concluded that idarubicin was stable for a period of 28 days. However, this work has limitations and lacks specific details regarding the criteria currently recommended for stability study procedures. [Bardin et al. 2012; SFPC/GERPAC 2013; ICH Q1A(R2) 2003] Indeed, this study was not conducted under routine clinical conditions of use, idarubicin having been diluted in polypropylene tubes and not in bags of diluents for infusion. Furthermore, fundamental criteria on physicochemical stability (determination of visible, sub-visible particles, osmolality, and pH monitoring) are missing or lack details. Moreover, idarubicin concentrations used in routine practice frequently deviate from the target value of 0.1 mg/mL studied or may be stored in the refrigerator by accident. Extrapolating data from stability studies to conditions other than those studied (e.g. concentrations, solvent, storage conditions) involves risks and engage the responsibility of the pharmacists who produce these preparations.”*

Bibliography :

Beijnen JH, Rosing H, De Vries Ph. A, Underberg WJM. Stability of anthracycline antitumour agents in infusion fluids. Journal of Parenteral Science and Technology. 1985 Nov 1;39(6):220–2. PubMed PMID: 3867751.

Bardin C, Astier A, Vulto A, Sewell G, Vigneron J, Trittler R, et al. Guidelines for the practical stability studies of anticancer drugs: a European consensus conference. 2012;278–85. doi:10.1136/ejhpharm-2012-000112

SFPC/GERPAC. Methodological guidelines for stability studies of hospital pharmaceutical preparations, Part I : Liquid preparations. [Internet]. 2013. Available from: http://www.gerpac.eu/IMG/pdf/%0Aguide_stabilite_anglais.pdf

ICH Q1A(R2). Stability Testing of New Drug Substances and Products. 2003; Available from: https://database.ich.org/sites/default/files/Q1A%28R2%29 Guideline.pdf

The Handbook of Injectable Drugs also identifies another stability study not referenced in your manuscript: Farmitalia Carlo Erba. Stability of 4 demethoxydaunorubicin hydrochloride reconstituted solutions with water for injections, sodium chloride, dextrose. Milan, Italy 1986. It might be interesting to analyze and include this work.

Thank you for the reference you provided. We would like to clarify that this reference is not a published scientific article, but it appears to be data from an internal study conducted by the pharmaceutical company Farmitalia.

After further research, we were able to access the following two sources: “Handbook on Injectable Drugs” (17th edition, 2013) and “ASHP Injectable Drug Information: A Comprehensive Guide to Compatibility and Stability” (2021 edition). The ASHP guide includes all the information contained in the Handbook (usually called Trissel’s).

<https://news.ashp.org/news/meetingnews/2023/04/03/how-does-ashp-injectable-drug-information-stack-up-against-other-resources?utm>).

We were able to compare the information in the section on idarubicin, which is exactly the same (see screenshots below).

Ref 1493: Farmitalia Carlo Erba. Stability of 4–demethoxydaunorubicin hydrochloride reconstituted solutions with water for injections, sodium chloride, dextrose, and sodium chloride with dextrose injections. Milan, Italy; 1986 Jun.

This reference states that (see the screenshoots below), in Dextrose 5% or in Sodium Chloride 0.9%, “no loss in 72 hr at room temperature protected from light. Less than 10% loss in 6 hr at room temperature exposed to light”.

These findings are consistent with the results of our study, which was conducted under “light-protected” conditions. While evaluating the stability-indicating capability of our HPLC method, we also observed this sensitivity to light.

For information, Farmitalia is a pharmaceutical group that has been acquired and merged with other companies, including Pfizer, which now markets Zavedos®.

<https://en.wikipedia.org/wiki/Farmitalia>

<https://www.pfizer.com/about/history/pfizer_pharmacia>

To date, the information in the summary of product characteristics for Zavedos® does not include the data provided by Farmitalia.

See: <https://agence-prd.ansm.sante.fr/php/ecodex/extrait.php?specid=66099467>

That is why we are wondering whether to cite the Farmitalia reference, given that this laboratory no longer exists and Pfizer does not include these data.

Idarubicine in Handbook on Injectable Drugs” (17th edition, 2013)

Idarubicine in “ASHP Injectable Drug Information: A Comprehensive Guide to Compatibility and Stability” (2021 edition).

Ref 1493: Farmitalia Carlo Erba. Stability of 4–demethoxydaunorubicin hydrochloride reconstituted solutions with water for injections, sodium chloride, dextrose, and sodium chloride with dextrose injections. Milan, Italy; 1986 Jun.

L 73: Does idarubicin not exist in standard SCR quality (EDQM pharmacopoeia)?

No, to the best of our knowledge, idarubicin is not available (<https://crs.edqm.eu/>).

L19: There is a generic drug in France. What are the differences, and can the data obtained be extrapolated between the different forms available?

According to our information, Accord Laboratories markets a generic version of Zavedos (see the screenshots below obtained from <https://www.theriaque.org/apps/recherche/rch_simple.php>).

The difference lies in the pH adjustment; the generic version of Accord contains additional sodium hydroxide compared to the brand-name version of Zavedos. Furthermore, the pH of the generic version can range from 3 to 4.5, while that of the brand-name version ranges from 3 to 4.

This difference seems minor and may not have any consequences, but since we have not evaluated this specialty, we cannot offer an opinion.

L106: Beyond purity, is there water in the powder?

Yes, the water content in the idarubicin we used was 2.1% (this detail has been added (line 150), and we took that into account in our calculations.

L106: The description of the Milli-Q water production system should be detailed earlier, for example at the production of mobile phases, as we imagine that this water was used in the preparation of phosphate buffer solutions.

This description has been added to the “Materials” section (between line 90 to 91)

L110: “a serie” and not “a series”

The typo has been corrected (Line 154)

L116: Were the QC points achieved with API powder from Sigma Aldrich?

Yes, the QC points were achieved with API powder.

Is there a monograph in the pharmacopoeia for idarubicin?

To date, there is no monograph in the European Pharmacopoeia. There is a monograph in the USP Pharmacopeia.

L140: Define the parameters that justify satisfactory separation at the peak level.

The following clarification has been added (between line 185 to 189):

*“All the chromatograms obtained were compared to the reference chromatograms obtained with the assay method described above, using samples not subjected to stress conditions.* ***The degradation parameters were optimized to ensure effective separation down to the baseline, or to minimize overlap between the peaks of the degradation products and the idarubicin peak.*** *All tests were performed in triplicate.”*

It is unusual to group the acceptance criteria in the same paragraph;

The acceptance criteria for chemical stability have been separated from those for pH, osmolarity, and particle count, which have been moved to their respective sections describing the methods used (between line 198 to 218).

I am surprised by the detection of degradation products as a criterion for non-acceptance;

We checked for the presence of degradation products but did not specify that this was a criterion for rejection. We understand the confusion this sentence may have caused and have removed this point (between line 191 to 193).

For pH and osmolarity, there could be a statistically significant difference without any repercussions, depending also on the number of samples and the power; it may therefore be more appropriate to set threshold values as specifications; for the pH specification, it is important to adapt it to the presence or absence of buffers in the preparation.

We have taken your comment into account and replaced our statistical analysis of pH with a criterion of a maximum variation of one unit (between line 202 to 203). With regard to osmolality, the statistical analysis was also removed.

For method optimization, it may be useful to determine the resolution coefficients;

We would like to thank the reviewer for this advice. Not all the results of the optimisation of the stability indicating method have been included in this article. We use an initial visual approach to peak separation to assess the effectiveness of each optimisation step. When we have to choose between two results obtained using different parameters, we naturally use that parameter.

Zoom on s2 poorly represented

The S2 figure represent the heating conditions. We have improved it and expect it will meet your expectation.

Deg 2 on s3: unresolved peak

The S3 figure represent the oxydative conditions. This experiment was repeated, and the results were improved (see response to editor page 3-8 of the present document).

Deg 3 and 4 S5: unresolved peaks

The S5 figure represent the alkaline conditions. This experiment was repeated, and the results were improved (see response to editor page 3-8 of the present document)

S6: the results show unresolved peaks and the retention time varied greatly; is there an explanation for this? Why not shorten the 24-hour time considerably in the hope that the degradation would not be too significant?

The S6 figure represent the light condition. As you can see in the response to the editor (page 3-8 of the present document), we have shorten the time of exposition and obtained a lower degradation rate.

For representation of stability over time: the points in time on the x-axis should be spaced appropriately.

In accordance with your recommendations, we have revised the figure as follows.

L191: the significant number of LOD and LOQ is too high and inappropriate.

We followed your recommendations and reduced the significant number of LOQ and LOD from 4 to 2 (between line 249 to 250).

L261: not “maintained” because n is not a pH buffer.

In fact, that word was inappropriate, so we changed it to “QS pH from 3 to 4” (Line 327)

L 263: Why is polyolefin the preferred format for chemotherapy bags? There is no bibliographic reference to support this assertion.

We have combined our response with another one of your questions above (page 12-13 of the present document).

L 270: The paragraph on osmolarity is not useful and contributes little to the discussion itself; in fact, the starting specialty is isotonic and placed in a larger isotonic volume, so it is not surprising to have an isotonic solution in the end.

This paragraph has been removed

L277 The particle values are very high at D0, particularly for glucose, and decrease thereafter. Is there an explanation or a causal reason for this phenomenon?

To the best of our knowledge, we have no explanation for this phenomenon.

You indicate that this may be related to the control environment not being sterile; however, did this change during subsequent measurements?

At the start of the study, we noticed this high particle count and immediately suspected external contamination or contamination of the glass vials into which the samples were transferred for counting by the instrument. This hypothesis is supported by the fact that the sample is exposed to the open-air during analysis rather than being placed in a closed system. For this reason, we took special precautions: no staff member was allowed to enter or exit the laboratory during the analyses, and staff movement around the equipment was restricted. Furthermore, particular attention was given to the preparation of the samples in the glass vials.

This is potentially related to an initial release of particles from the material, but this has not been discussed; did you test several batches of bags?

No, we did not test multiple batches. Each bag was measured on days 0, 15, and 30. Before each measurement, the bags were shaken before samples were taken. We assume that if particle release had been the cause of this contamination, the number of particles detected on days 15 and 30 would have been higher.

Despite this unexplained contamination, the results remain in line with the requirements of the pharmacopoeias.

L280 What was the assay method used in this study and was it stability indicating?

No, the information provided in this article does not allow the HPLC method used to be classified as a stability indicating. This point has been clarified (Line 349).

L 283 I find the method for determining the concentrations tested useful with the explanation; I think it could be included in the introduction or materials and methods section?

Thank you for your comment. We have added a section in material and method section to explain the methodology of this preliminary study in detail to our readers (Line 96-109).

***“Choice of concentrations for the stability study***

*To guide the choices of concentrations for the stability study, a preliminary analysis of injectable anticancer drug production data from University Hospital of Bordeaux was conducted. The data were extracted from Chimio® production management software (Computer Engineering, Paris, France) and were centered on idarubicin production in bags between 2016 and 2023 intended for adult and pediatric oncohematology care units.*

*University Hospital of Bordeaux makes approximately 80,000 injectable preparations per year. Over the study period, 2,172 idarubicin bags were made as part of acute lymphoid leukemia, acute myeloid leukemia and lymphoma treatment regimens (Figure S8). Preparations intended for adults represented over 95 % of production. As the dose banding approach was not applied for idarubicin dose standardization, 23 different concentrations between 0.02 and 0.28 mg/mL were produced, of which close to 87 % were centered between 0.09 and 0.14 mg/mL. The limits of the stability study were defined between 0.04 mg/mL and 0.2 mg/mL, with concentrations beyond these limits concerning fewer than 10 preparations over the 8 years of analysis.”*

# Response to reviewer 2:

The study is well conducted and results are very usefull for hospital pharmacists

I recommend the publication of the article after a minor review.

We would like to thank the reviewer for their interest in our work.

Kindly find below my comments and recommendation

Line 70: bags were… It would be better to formulate as : For solution dilution, Freeflex bags were purchased from … The infusion bags were made of…..

As requested, the wording has been changed as follow:

*“For solution dilution, Freeflex+® bags were purchased from Fresenius Kabi (*[*Bad Homburg vor der Höhe, Germany*](https://www.google.com/search?client=firefox-b-d&q=bad+homburg&stick=H4sIAAAAAAAAAONgVuLUz9U3MMouyjN_xGjCLfDyxz1hKe1Ja05eY1Tl4grOyC93zSvJLKkUEudig7J4pbi5ELp4FrFyJyWmKGTk5yaVFqUDACcrq21SAAAA)*). The infusion bags were made of polyolefin (primary internal film: polypropylene; secondary external film: polypropylene/polyethylene admixture).”*

Line 80-82: it can be clearer for the reader if short sentences are used

A parenthesis was missing from the manuscript

We suggest simplifying the sentence as follows:

*Three variation factors were studied and crossed: the diluent (0.9 % NaCl or 5 % Dex), the final concentration (0.04 mg/mL or 0.2 mg/mL) and the storage temperature (room temperature 22 ± 3 °C or refrigerated conditions 5 ± 3 °C).*

We hope this makes the sentence clearer (see line 113 to 116).

Line 118: same instead of sale

The typo has been corrected.

Line 202: a table with results and the mass balance for each condition should be added and table 1 should give more information

The following table (Table 2: results of the forced degradation study) has been added (line 263).

| Type of degradation | Conditions | Percentage of degradation | Mass balance (%) |
| --- | --- | --- | --- |
| Heating stress | 80 °C | 38.2 % 4 h | 60,8 |
| Oxidative | H_2_0_2_ 7.5 % | 22.7 % - 30min | 89,1 |
| Acidic | HCl 0.1 M – 80 °C | 34.3 % - 5 min | 95,2 |
| Alkaline | NaOH 1 M – 70 °C | 15.2 % - 1 min | 86,8 |
| Photolytic stress | UV – 20 °C | 19.3 % - 2h | 87,1 |

In accordance with your recommendations, the mass balance has been included. As a reminder, the principle is to check that the loss of the main compound is consistent with the appearance of degradation products. It was calculated by comparing the area under the idarubicin reference peak with the sum of the areas under the remaining idarubicin peak and the peaks of the degradation products, and expressed in percentage.

Ideally, this value is close to 100%. In practice, a recovery rate of between 95 and 105% is often considered satisfactory (see SFPC/GERPAC. Methodological guidelines for stability studies of hospital pharmaceutical preparations, Part I : Liquid preparations. [Internet]. 2013. Available from: <http://www.gerpac.eu/IMG/pdf/%0Aguide_stabilite_anglais.pdf>).

However, with HPLC-UV/Vis detection, this recovery is approximate, because: degradation products may have UV absorption coefficients that differ from that of the main compound; some products may not absorb at the selected wavelengths. In our context and in view of the intended objectives, the results achieved meet our requirements.

Line 207; 209; 2016: degradation products should be named differently for exemple DEG1 and DEG2 for the S2 fig then DEG3 and DEG4 for the S3fig … as they are not the same.

We have taken this comment into account (see response to the editor page 4 of the present document)

Are degradation products described in the literature or in pharmacopoeia?

There is no monograph in the European Pharmacopoeia (V 12.1). A monograph is available in the USP (43 NF 38) but does not mention any degradation products.

Line 231: is the concentration of the sample enough to detect degradation product? We usually use more concentrated solution for that. There is no information about the sample solution. Are solutions analyzed without any dilution? And at this concentration, has the limit of detection of degradation products been tested? What are the specifications for degradation products? Such information should be given for stability indicating method. The limits have been specified only for the assay for the active substance

Our manuscript lacks details regarding sample processing. These details (whether or not the samples were diluted) have been added (between line 169 to 189).

We believe that the concentrations used in this study were sufficient to detect degradation products. In fact, the limit of detection for idarubicin was estimated to be 6.01 × 10⁻⁵ mg/mL.

During forced degradation study, one mL of 0.04mg/mL idarubicine solution were either analyzed without dilution after heating and light exposition, diluted by half in oxidative conditions or diluted to one-third in alkalin and acidic exposition. Thus, a 0.04 mg/mL sample diluted to one-third of its original concentration will be 250 times more concentrated than the detection limit.

Under these conditions, degradation products were detected and their concentrations can be estimated. For example, the product named DEGL7 (Fig S6 for light degradation) is one of the degradation products observed to have the lowest AUC amongst our five test conditions. Its concentration was estimated at 3.6 × 10⁻³ mg/mL, which is still 60 times higher than the detection limit of our method.

This approach is in fact based on the assessment of the detection limit of native idarubicin, rather than that of the degradation products themselves. This would, on the one hand, require knowledge of the exact formula of the degradation products. This is not possible with the DAD detection method. Furthermore, as these molecules have not been previously characterised, it is not possible to compare their spectra with a catalogue of known molecules. Moreover, this would require these molecules to be available in their native form from a chemical supplier, in order to assess their detection and quantification limits using our method, which is not the case. We are therefore unable to provide any further information regarding the details requested.

The aim of the study was to investigate the stability of idarubicin under clinical conditions. We consider an idarubicin preparation to be stable if its concentration varies between 90 and 110 per cent of the target concentration. Generally speaking, the formation of degradation products is a sign of instability. The appropriate course of action in their presence must be assessed in light of the quantity detected and their toxicity, where such information is available. We evaluated whether our HPLC assay method would be capable of detecting degradation products under our study conditions. Under these conditions, we consider the limit of detection and the results obtained to be satisfactory.

On an exploratory basis, there is always scope to improve the detection of degradation products. This could involve, for example, using more sensitive detection methods such as mass spectrometry. It is also possible to work with highly concentrated solutions in order to study a molecule’s degradation processes in greater detail. Then, in a second stage, the detection of these molecules at lower concentrations could be explored. This approach could provide more information on the degradation products. However, although this approach is very interesting from a scientific point of view, it would stray somewhat from the practical objective originally set.

Line 239: and for the dextrose bags?

This was an error on our part during the revision of our article. The pH of the dextrose-based preparations was also stable, with variations of less than one unit.

The sentence was modified as follow (line 303):

“*For the pH measurements on days D0, D15 and D30, in the NaCl* ***0.9% and Dex 5%*** *bags, no variation greater than one pH unit was observed compared to the pH at the start of the study (D0) (S2 Table, Fig 2) independently of temperature.”*

# Response to Reviewer 3:

Article review for PLOS One:
One-month physicochemical stability study of idarubicin prepared in polyolefin bags for the treatment of hematological diseases
2026-03-10

GENERAL:
Good morning! It's a pleasure to read your work. A very interesting and well-written article.

We would like to thank Reviewer 3 for his encouraging feedback

SPECIFIC:
Excellent article, requiring very few changes. Could you add a discussion on the microbiological aspects you haven't addressed, which might limit its usefulness? This microbiological aspect is the only thing missing from your article

*As a group of experts has described (Bardin et al. 2012, Crauste-Manciet et al. 2020), the shelf-life should be validated taking chemical, physical and microbiological data into consideration.*

*Microbiological stability depends on the preparation's ability to support the growth of microorganisms. As demonstrated by Krämer in 1998, idarubicin has some antimicrobial activity, but this may be limited against some microorganisms (bactericidal effect at 0.07mg/mL after 120 hours of incubation on Staphylococcus Aureus, Enterococcus Faecium and Candida Albicans but Pseudomonas Aeruginosa remains viable). The risk of persistent microbiological contamination is therefore not zero, and this risk must be evaluated taking other important factors into account.*

*In fact, microbiological stability depends also on the preservation of sterility in the final container which much be evaluated by microbiological and physical integrity tests (NHS 2013).*

*Finally, the initial sterility of the preparation may involve the application of Good Manufacturing Practices and are very closely linked to the practices of centralized units: production process, ISO 5 equipment used, closed systems used, adding of a clamped infusion set to the bags or not, operator training and validation, disinfection procedure, secondary packaging used, storage conditions etc… For these reasons, it is recommended to conduct a risk assessment of the preparation and storage process; to regularly validate the preparation process (process simulation tests with culture media), the staff members (conducting media fill tests) and the equipment (microbiological performance qualification).*

Bibliography:

Bardin C, Astier A, Vulto A, Sewell G, Vigneron J, Trittler R, et al. Guidelines for the practical stability studies of anticancer drugs: a European consensus conference. 2012;278–85. doi:10.1136/ejhpharm-2012-000112

Crauste-Manciet S, Krämer I, Lagarce F, Sautou V, Beaney A, Smith J, et al. GERPAC Consensus Conference – Guidance on the Assignment of Microbiological Shelf-life for Hospital Pharmacy Aseptic Preparations. Pharm Technol Hosp Pharm. 2020 May 26;5(1). doi:10.1515/pthp-2020-0001

Irene Krämer. Viability of microorganisms in novel antineoplastic and antiviral drug solutions. Journal of Oncology Pharmacy Practice. 1998;4(1):32–7. doi:ttps://doi.org/10.1177/10781552980040010

<https://sps.nhs.uk/wp-content/uploads/2013/07/NHS-PQAC-YCD-Integrity-Testing-of-Syringes-2013-2nd-Edn1.pdf>

SUMMURY:

• I don't quite understand the second sentence: "The literature lacks… stability studies." Could you clarify your point?

After reviewing it, we believe that the following revised version of the sentence would clear up any confusion: (line 22) “The literature lacks **stability data** that comply with current preparation practices and recommendations for conducting stability studies”.

As we explain in more detail in the introduction, there are few stability studies in the literature concerning idarubicin diluted in a bag. The only published study available to us is that by Beijnen et al. from 1986. This work has limitations and lacks specific details regarding the criteria currently recommended for stability study procedures (Bardin 2012; SFPC/Gerpac 2013).

Bardin C, Astier A, Vulto A, Sewell G, Vigneron J, Trittler R, et al. Guidelines for the practical stability studies of anticancer drugs: a European consensus conference. 2012;278–85.

SFPC/GERPAC. Methodological guidelines for stability studies of hospital pharmaceutical preparations, Part I : Liquid preparations. [Internet]. 2013. Available from: http://www.gerpac.eu/IMG/pdf/%0Aguide_stabilite_anglais.pdf

As recommended by Reviewer 1, we have expanded on the limitations of the Beijnen et al. study in the introduction (line 58-71)

• Usually, we don't start a sentence with "pH" but rather "The pH…" or something similar.

The correction has been made (line 35).

INTRODUCTION

• Page 3, line 49. You didn't also conduct the stability study in syringes? Wouldn't that have been relevant?

Thank you for your insightful comment. In fact, since the summary of product characteristics does not specify otherwise, the manufacturer leaves the choice of the final container up to the healthcare team. This can be a bag or a syringe.

In our center, bags are used for several reasons: clinical (increased patient hydration), practical for pharmacy technicians (fewer steps required to prepare the medication), practical for nurses (does not require an infusion pump), and cost-effective (reduced amount of supplies needed for preparation compared to a syringe). These practices align with those followed by reference centers similar to ours in size and organization and explain the context in which this study was conducted.

As we noted in the introduction, the purpose of this study is to examine the stability of idarubicin in hematological indications. To our knowledge, in these indications, idarubicin is administered only in diluted form from a bag, in accordance with the summary of product characteristics (SPC).

• Page 3, line 64: Why not use PVC bags anyway? They are the most commonly used for all treatments in North America. But you explain later that this isn't the case for chemotherapy in Europe…

We have combined our response to your question with Reviewer 1’s response on the same topic (see page 12 of the present document).

METHODS
• Page 4, lines 73 to 77: Shouldn't the lot numbers of each ingredient be included at a minimum?

We do not usually record the batch numbers of common laboratory consumables such as acetonitrile and other salts that we use frequently. In light of your comment, this appears to us to be a shortcoming in our practices. We will rectify this for our future studies.

However, we do have the batch numbers for the analytical reference idarubicin (batch 0000424922 , exp: 2024 11) and the Zavedos product (batches CA7906 exp 2022 03 and DX0725 exp 2023 03). We are adding these to the manuscript. Note that, we would like to point out that the initial experiments for this study were carried out in March 2020.

• Page 5, line 107: Is it normal to have a packet of "X"s after "PF1"?

The retailer (from whom we purchased the device) part number is PF1XXXXM1 (see: [https://www.fishersci.fr](https://www.fishersci.fr/shop/products/videos/12083273)). The manufacturer's part number is Purelab Flex 1 (see: [https://www.elgalabwater.com](https://www.elgalabwater.com/purelabr-flex-1?_gl=1*1sncl5t*_up*MQ..*_gs*MQ..*_ga*MzM1Mzg5MjU1LjE3NzM3NjU4NjE.*_ga_97KB2EN3XY*czE3NzM3NjU4NjEkbzEkZzAkdDE3NzM3NjU4NjEkajYwJGwwJGgw*_ga_DBGXDYTJ1T*czE3NzM3NjU4NjEkbzEkZzAkdDE3NzM3NjU4NjEkajYwJGwwJGgw&gclid=Cj0KCQjw9-PNBhDfARIsABHN6-1OE_uhcC4cncWXFlJfL9WxtKB5_3rshGaJffDKtEVRZTZVkqPaQ8MaAt9CEALw_wcB&gclsrc=aw.ds)). For clarity, we have updated the information to include the laboratory's reference number (line 151).

• Page 5, line 118: What exactly is a "sale assay"?

It’s a typo. The correct word is “same”.

The sentence was modified by : “The interday repeatability (precision and accuracy) was determined by repeating the **same** assay on 3 different days” (line 162).

RESULTS
• Page 8, line 198: Is it normal for the bottom right-hand cell in the table to be empty?

Yes, it is normal not to have an equation in the case of interday analysis. This is because, for intraday analysis, each range is obtained from six independent samples and repeated three times. For each range, a straight-line equation is calculated. In interday analysis, all these results are compared with one another to assess the accuracy of the method.

• Page 9, lines 207, 208, and 216: Why reuse the same numbering, DEG1 and DEG2? Are we still talking about the same data? Or should the numbering be sequential for clarity?

We understand the confusion this may have caused. For clarity, we have renamed the degradation products. We have combined our response to your question with Editor’s response on the same topic (see page 4 of the present document).

• Page 9, line 219: So, is using an amber bag mandatory?

Yes, we recommend storing the product in a container that protects it from light. Light sensitivity was also noted by Bourcier et al., who conducted their stability study in syringes protected from light. Furthermore, as explained in the response to Reviewer 1’s question, anthracyclines are known to be sensitive to light. This clarification was added to the manuscript (line 408-414).

• Page 10, line 234: Shouldn't the "V" in the temperature line represent the temperature?

No, it’s a typo. The table 2 has been corrected with the right temperature: 22 ± 3 °C.

• Page 10, lines 234 and 235: You repeat "OC OC" three times.

It’s a typo. The table 2 has been corrected.

• Page 10, lines 239 to 242: And what happens with the 5% dextrose?

This was an error on our part during the revision of our article. The pH of the dextrose-based preparations was also stable, with variations of less than one unit.

The sentence was modified as follow (line 303-305):

“*For the pH measurements on days D0, D15 and D30, in the* ***0.9% NaCl and 5% Dex*** *bags, no variation greater than one pH unit was observed compared to the pH at the start of the study (D0) (S2 Table, Fig 2) independently of temperature.”*

• Page 10, lines 243 to 245: This key is not below its figure. Here again, do not begin a sentence directly with "pH".

We apologize for any inconvenience this may cause but, in accordance with the recommendations of PLOS ONE, the “Figure captions are inserted immediately after the first paragraph in which the figure is cited. Figure files are uploaded separately.”

As recommended, the Figure 2 title was modified as follow: “**Measurements of** pH (A, B, C, D) and osmolarity (E, F,) over time of idarubicin diluted at 0.04 mg/mL and 0.2 mg/mL in 0.9% NaCl and 5% Dex IV bags” (line 307).

• Page 11, lines 246 to 258: Do these criteria apply to both sodium chloride and 5% dextrose?

Yes, for both conditions. For clarity we modified the sentence as follow (line 311-317):

*Similarly,* ***osmolality of the 0.9% NaCl and 5% Dex bags*** *on days D0, D15 and D30 remained stable at around 5 units on average compared with the osmolality on D0 (S3 Table, Fig 2). These differences in osmolality observed were not significant between D0, D15 and D30 (P value: P > 0.05).*

*For the particle count on days D0, D15 and D30, the stability study demonstrated compliance with European Pharmacopoeia guidelines* ***in both 0.9% NaCl and 5% Dex bags****. The number of particles measured remained below 6,000 per container for particle ≥10 µm in size of and below 600 per container for particle ≥25 µm in size (S4 Table; Fig 3).*

• Page 11, lines 253 to 256: This key is not below its figure.
As with your comment on Figure 2, we followed the journal’s recommendations

DISCUSSION
• Page 12, line 283: Usually, we avoid referring to "our study" and instead use "the present study," as you have done elsewhere.

As recommended, the sentences were modified as follow

Line 325: “***The present study*** *focused on the brand-name drug…”*

Line 353: *“The* ***results of the present study*** *are consistent with….”*

Line 372: “In the ***context of the present study****….*”

• Page 12, line 287: Is it "g/ml" or mg/ml?

It’s a typo. The unit is in mg/ml.

• Page 13, line 313: Here again, we avoid referring to "our study" and instead use "the present study," as you have done elsewhere.

As recommended, the sentences was modified.

CONCLUSION
Correct in itself, very judicious.

REFERENCES:
Correct

APPENDICES:
Correct

Once again, we would like to thank the reviewer for their precise and insightful analysis.

# Request for an extension of the review period

Dear Dr Bouguéon,

Thank you for your recent message. We received your request to extend the resubmission date for your revised manuscript. It is perfectly fine to have an extension in order to complete your revisions. I have extended your due date to September 12, 2026. 

We can keep manuscript files open six months since the editorial decision was issued.

If you anticipate your revisions will take longer than six months, we would still be happy to consider the work, but in those circumstances we would recommend that the revised manuscript is submitted as a new submission to PLOS ONE – as we would have to close the file for the original submission if a revision is not received within six months.

Should you choose to resubmit as a new submission, please refer to the original manuscript number in the cover letter.

We do hope you will be able to complete the revisions within the outlined turnaround but if you do not think that will be possible, please do let us know so that we can take the necessary steps regarding the submission file.

Please feel free to contact us if you have any questions. We look forward to seeing your revised manuscript.

Kind regards,
Roxanne Jastine Baltar
Straive Editorial Assistant

PLOS ONE | plosone@plos.org
Empowering researchers to transform science

Case Number: 09598545
thread::ZDyrFqRQfjuJWYd19CwB1Bo::

Original Message ---------------

From: Guillaume Bougueon [guillaume.bougueon@u-bordeaux.fr]

Sent: 4/16/2026, 1:14 AM

To: plosone@plos.org

Cc: jean-marc.bernadou@chu-bordeaux.fr; fabien.xuereb@chu-bordeaux.fr

Subject: Re: PLOS One: Revision Reminder (PONE-D-26-07865) - [EMID:ac7540ec68ef3898]

EXTERNAL EMAIL – SECURITY NOTICE

This message originated outside of PLOS. Exercise caution when interacting with links, attachments, or requests for sensitive information.

[You don't often get email from guillaume.bougueon@u-bordeaux.fr. Learn why this is important at https://aka.ms/LearnAboutSenderIdentification ]

EXTERNAL EMAIL – SECURITY NOTICE

This message originated outside of PLOS. Exercise caution when interacting with links, attachments, or requests for sensitive information.

Dear member of the PLOS ONE team,

Thank you for your follow-up email.

The revision of our article is currently underway, and additional revisions have been made.

In order to refine our work and allow all authors to approve the final version, we would need some additional time.

Would it be possible to grant us this extension?

Please let us know if you need any further information.

Have a nice day,

Best regards

Guillaume BOUGUÉON

Pharmacien, Maître de Conférences des Universités-Praticien Hospitalier

Laboratoire ARNA, ChemBioPharm, Groupe BANCO, U1212 INSERM - UMR 5320 CNRS ,

Université de Bordeaux Collège et département de sciences de la santé - UFR Sciences pharmaceutiques site Carreire 146, rue Léo Saignat 33076 Bordeaux Tel:+33(0)5.57.57.46.96

guillaume.bougueon@u-bordeaux.fr

guillaume.bougueon@inserm.fr

Pharmacie Groupe Hospitalier Sud ,

Hôpital Haut-Lévêque, CHU de Bordeaux,

Avenue de Magellan 33604 Pessac, Tel: +33(0)5.57.65.64.95

guillaume.bougueon@chu-bordeaux.fr
