# Supplementary material for: One-month physicochemical stability study of idarubicin prepared in polyolefin-type bags for the treatment of hematological diseases
Source: PLoS One. 2026 Aug 28;21(8):e0356906. doi: 10.1371/journal.pone.0356906 (PMC13524237; doi:10.1371/journal.pone.0356906)
Supplement: Peer review history

# Peer review: Decision Letter 0

**DOI:** 10.1371/journal.pone.0356906.r001

**Authors:** Christophe Curti

12 Mar 2026

Dear Dr. Bouguéon,

Thank you for submitting your manuscript to PLOS ONE. After careful consideration, we feel that it has merit but does not fully meet PLOS ONE’s publication criteria as it currently stands. Therefore, we invite you to submit a revised version of the manuscript that addresses the points raised during the review process.

Please submit your revised manuscript by Apr 26 2026 11:59PM. If you will need more time than this to complete your revisions, please reply to this message or contact the journal office at plosone@plos.org. When you're ready to submit your revision, log on to https://www.editorialmanager.com/pone/ and select the 'Submissions Needing Revision' folder to locate your manuscript file.

- A letter that responds to each point raised by the academic editor and reviewer(s). You should upload this letter as a separate file labeled 'Response to Reviewers'.
- A marked-up copy of your manuscript that highlights changes made to the original version. You should upload this as a separate file labeled 'Revised Manuscript with Track Changes'.
- An unmarked version of your revised paper without tracked changes. You should upload this as a separate file labeled 'Manuscript'.

If you would like to make changes to your financial disclosure, please include your updated statement in your cover letter. Guidelines for resubmitting your figure files are available below the reviewer comments at the end of this letter.

If applicable, we recommend that you deposit your laboratory protocols in protocols.io to enhance the reproducibility of your results. Protocols.io assigns your protocol its own identifier (DOI) so that it can be cited independently in the future. For instructions see: https://journals.plos.org/plosone/s/submission-guidelines#loc-laboratory-protocols. Additionally, PLOS ONE offers an option for publishing peer-reviewed Lab Protocol articles, which describe protocols hosted on protocols.io. Read more information on sharing protocols at https://plos.org/protocols?utm_medium=editorial-email&utm_source=authorletters&utm_campaign=protocols.

We look forward to receiving your revised manuscript.

Kind regards,

Christophe Curti

Academic Editor

PLOS One

Journal Requirements:

When submitting your revision, we need you to address these additional requirements.

1. Please ensure that your manuscript meets PLOS ONE's style requirements, including those for file naming. The PLOS ONE style templates can be found at

https://journals.plos.org/plosone/s/file?id=wjVg/PLOSOne_formatting_sample_main_body.pdf and

https://journals.plos.org/plosone/s/file?id=ba62/PLOSOne_formatting_sample_title_authors_affiliations.pdf

2. When completing the data availability statement of the submission form, you indicated that you will make your data available on acceptance. We strongly recommend all authors decide on a data sharing plan before acceptance, as the process can be lengthy and hold up publication timelines. Please note that, though access restrictions are acceptable now, your entire data will need to be made freely accessible if your manuscript is accepted for publication. This policy applies to all data except where public deposition would breach compliance with the protocol approved by your research ethics board. If you are unable to adhere to our open data policy, please kindly revise your statement to explain your reasoning and we will seek the editor's input on an exemption. Please be assured that, once you have provided your new statement, the assessment of your exemption will not hold up the peer review process.

3. If the reviewer comments include a recommendation to cite specific previously published works, please review and evaluate these publications to determine whether they are relevant and should be cited. There is no requirement to cite these works unless the editor has indicated otherwise.

**Additional Editor Comments:**

Dear Authors,

According to reviewers reports, I recommend you (in addition to your point-to point answer to reviewers comments) to improve the forced degradation experiments part as reported by all reviewers. Reviewer 1 have identified several flaws which probably require additionnal experiments.

From my point of view, and after reading this part:

For light and alkaline degradation experiments, idarubicine was too much degraded. Generally, during forced degradation experiments, a degradation approximately equal to 10-30% is targeted to identify primary degradation products and to obtain a well-resolved mixture. Light and alkaline experiments should be probably done again under new conditions.

For oxidative experiment, an unresolved peak is observed (DEG 2, RT 2.707). It appears as a limitation of your work unless new data are provided. Moreover, two peaks were reported to be related to H2O2 (2.707 and 4.300)? Are you sure? H2O2 in excess is not the first big peak with RT 2.0 min?

For light experiment (S6), why is there a "NaOH" peak at 1.873 min?

Improve degradation products numbering as requested by reviewers 2 and 3 (don't reuse number). Moreover, I recommend you to provide RRT rather than retention time, and you could also identify degradation products which appear under several degradation conditions.

[Note: HTML markup is below. Please do not edit.]

Reviewers' comments:

Reviewer's Responses to Questions

**Comments to the Author**

1. Is the manuscript technically sound, and do the data support the conclusions?

Reviewer #1: Partly

Reviewer #2: Yes

Reviewer #3: Yes

**********

2. Has the statistical analysis been performed appropriately and rigorously?

Reviewer #1: N/A

Reviewer #2: Yes

Reviewer #3: Yes

**********

3. Have the authors made all data underlying the findings in their manuscript fully available?

The PLOS Data policy

Reviewer #1: Yes

Reviewer #2: Yes

Reviewer #3: Yes

**********

4. Is the manuscript presented in an intelligible fashion and written in standard English?

Reviewer #1: Yes

Reviewer #2: Yes

Reviewer #3: Yes

**********

Reviewer #1: Dear editor,

Thank you for asking me to review the manuscript “One-month physicochemical stability study of idarubicin prepared in polyolefin bags for the treatment of hematological diseases.”

This work involves determining the stability of idarubicin bag preparations. The data available to date are outdated and poorly documented. Stability data make it possible to plan preparations in advance and improve the use of these drugs in healthcare settings.

I find that the study does not analyze, or analyzes only to a limited extent, the factors causing degradation, particularly in the discussion section, which does not adequately describe the chemical phenomena that may occur in terms of molecule degradation.

Given the results of the forced degradation study, the data obtained are not very informative about the measures to be taken and the conditions to be avoided in order to prevent degradation of the molecule.

In the absence of data, it would also have been interesting to explore the stability of drugs with similar chemical structures in order to potentially predict the mechanisms of degradation of the molecule. Compared to other anthracyclines, do we see the same degradation profiles? And with the same effect depending on the material, concentration, and solvent?

I understand that there was no degradation of any kind during the stability study.

Regarding the chromatogram results: were there spectra with different wavelengths with the DAD? Because it is surprising that no degradation was found.

If no signs of degradation were found, why not conduct a longer stability study in order to characterize any potential degradation (and, moreover, store the product for longer)?

Furthermore, the term “polyolefin” is likely to mislead the reader, considering that all types of polyolefin were tested in contact with the molecule of interest, which is not what was done: only one free flex + model was tested, with polypropylene in contact with the molecule of interest. Why not test other materials (PVC, EVA, etc.)?

Finally, as a general comment, I did not find in the manuscript whether a specific protocol was implemented to protect the operators who carried out the control and stability study (handling of samples, risk of environmental contamination, etc.).

Minor corrections:

In the title: is it really necessary to specify “for the treatment of hematological diseases”?

P1 Not all authors have included their affiliation.

In Figure 2: how can we explain the decrease in concentration on Day 15 at a concentration of 0.04 mg/mL at 20°C, which we do not see subsequently?

L 57: provide a little more detail on the findings of the Beijnen stability study. The Handbook of Injectable Drugs also identifies another stability study not referenced in your manuscript: Farmitalia Carlo Erba. Stability of 4 demethoxydaunorubicin hydrochloride reconstituted solutions with water for injections, sodium chloride, dextrose. Milan, Italy 1986. It might be interesting to analyze and include this work.

L 73: Does idarubicin not exist in standard SCR quality (EDQM pharmacopoeia)?

L19: There is a generic drug in France. What are the differences, and can the data obtained be extrapolated between the different forms available?

L106: Beyond purity, is there water in the powder?

L106: The description of the Milli-Q water production system should be detailed earlier, for example at the production of mobile phases, as we imagine that this water was used in the preparation of phosphate buffer solutions.

L110: “a serie” and not “a series”

L116: Were the QC points achieved with API powder from Sigma Aldrich?

Is there a monograph in the pharmacopoeia for idarubicin?

L140: Define the parameters that justify satisfactory separation at the peak level.

It is unusual to group the acceptance criteria in the same paragraph; I am surprised by the detection of degradation products as a criterion for non-acceptance; For pH and osmolarity, there could be a statistically significant difference without any repercussions, depending also on the number of samples and the power; it may therefore be more appropriate to set threshold values as specifications; for the pH specification, it is important to adapt it to the presence or absence of buffers in the preparation.

For method optimization, it may be useful to determine the resolution coefficients;

Zoom on s2 poorly represented

Deg 2 on s3: unresolved peak

Deg 3 and 4 S5: unresolved peaks

S6: the results show unresolved peaks and the retention time varied greatly; is there an explanation for this? Why not shorten the 24-hour time considerably in the hope that the degradation would not be too significant?

For representation of stability over time: the points in time on the x-axis should be spaced appropriately.

L191: the significant number of LOD and LOQ is too high and inappropriate.

L261: not “maintained” because n is not a pH buffer.

L 263: Why is polyolefin the preferred format for chemotherapy bags?

There is no bibliographic reference to support this assertion.

L 270: The paragraph on osmolarity is not useful and contributes little to the discussion itself; in fact, the starting specialty is isotonic and placed in a larger isotonic volume, so it is not surprising to have an isotonic solution in the end.

L277 The particle values are very high at D0, particularly for glucose, and decrease thereafter. Is there an explanation or a causal reason for this phenomenon?

You indicate that this may be related to the control environment not being sterile; however, did this change during subsequent measurements? This is potentially related to an initial release of particles from the material, but this has not been discussed; did you test several batches of bags?

L280 What was the assay method used in this study and was it stability indicating?

L 283 I find the method for determining the concentrations tested useful with the explanation; I think it could be included in the introduction or materials and methods section?

Reviewer #2: The study is well conducted and is very usefull for hospital pharmacists. The results can be used by other hospital pharmacy to allow preparation in advance of Idarubicine.

I recommend the publication of the article after a minor review.

Kindly find below my comments and recommandation

Line 70 : bags were… It would be better to formulate as : For solution dilution, Freeflex bags were purchased from … The infusion bags were made of…..

Line 80-82: it can be clearer for the reader if short sentences are used

Line 118: same instead of sale

Line 202: a table with results and the mass balance for each condition should be added and table 1 should give more information

Line 207; 209; 2016: degradation products should be named differently for exemple DEG1 and DEG2 for the S2 fig then DEG3 and DEG4 for the S3fig … as they are not the same.

Are degradation products described in the literature or in pharmacopoeia?

Line 231: is the concentration of the sample enough to detect degradation product? We usually use more concentrated solution for that. There is no information about the sample solution. Are solutions analyzed without any dilution? And at this concentration, has the limit of detection of degradation products been tested? What are the specifications for degradation products? Such information should be given for stability indicating method. The limits have been specified only for the assay for the active substance

Line 239: and for the dextrose bags?

Reviewer #3: GÉNÉRAL :

Bonjour ! Une joie de vous lire. Article très intéressant et très bien monté. Et il me fait plaisir de vous adresser mes commentaires en français pour ensuite utiliser Google Translate pour que l’éditeur puisse comprendre… !

SPÉCIFIQUE :

Très bon article, ne requiert que très peu de modifications. Pourriez-vous ajouter une discussion sur les aspects microbiologiques que vous n’avez pas abordés et qui pourraient limiter votre date d’utilisation ? Ce côté microbiologique est le seul manquant à votre article.

SUMMURY :

• Je ne comprends pas bien la deuxième phrase : « The literature lacks… stability studies ». Pourriez-vous préciser votre idée ?

• Habituellement, on ne débute pas une phrase par « pH » mais bien plutôt « The pH… » ou quelque chose du genre.

INTRODUCTION

• Page 3 ligne 49. Vous n’avez pas mené l’étude de stabilité également en seringue ? Cela n’aurait pas été pertinent ?

• Page 3 ligne 64 : pourquoi ne pas avoir malgré tout utilisé des sacs de PVC ? Ce sont les plus utilisés d’habitude pour l’ensemble des traitements en Amérique du nord. Mais vous expliquez plus loin que ce n’est pas le cas pour la chimio en Europe …

METHODS.

• Page 4 lignes 73 à 77 : ne doit-on pas minimalement ajouter les numéros de lot de chacun des ingrédients ?

• Page 5 ligne 107 : c’est normal ce paquet de « X » après « PF1 » ?

• Page 5 ligne 118 : C’est quoi exactement un « sale assay » ?

RESULTS

• Page 8 ligne 198 : case du tableau en bas à l’extrême droite, c’est normal qu’elle soit vide ?

• Page 9 ligne 207, 208 et 216 : pourquoi avoir réutilisé la même numérotation soit DEG1 et DEG2 ? Parle-t-on toujours des mêmes données ? Ou faudrait-il une numérotation qui se suit pour plus de clarté ?

• Page 9 ligne 219 : donc l’utilisation d’un sac ambré est une obligation ?

• Page 10 ligne 234 : Le « V » à la ligne de température ne devrait-il pas être une température justement ?

• Page 10 ligne 234 et 235 : Par trois fois, vous répétez « OC OC ».

• Page 10 ligne 239 à 242 : Et qu’arrive-t-il avec le dextrose 5% ?

• Page 10 ligne 243 à 245 : cette légende n’est pas sous sa figure. Ici encore, ne pas commencer une phrase directement par « pH ».

• Page 11 lignes 246 à 258 : ces critères s’appliquent-ils tant pour le chlorure de sodium que le dextrose 5% ?

• Page 11 lignes 253 à 256 : cette légende n’est pas sous sa figure

DISCUSSION

• Page 12 ligne 283 : habituellement, on évite de parler de « notre étude » mais bien plutôt de « la présente étude » comme vous l’avez fait ailleurs.

• Page 12 ligne 287 : S’agit-il bien « g/ml » ou de mg/ml ?

• Page 13 linge 313 : Ici encore, on évite de parler de « notre étude » mais bien plutôt de « la présente étude » comme vous l’avez fait ailleurs.

CONCLUSION

Correcte en elle-même, très judicieuse.

REFERENCES :

Correctes

ANNEXES :

Correctes

Article review for PLOS One:

One-month physicochemical stability study of idarubicin prepared in polyolefin bags for the treatment of hamatological diseases

2026-03-10

GENERAL:

Good morning! It's a pleasure to read your work. A very interesting and well-written article.

SPECIFIC:

Excellent article, requiring very few changes. Could you add a discussion on the microbiological aspects you haven't addressed, which might limit its usefulness? This microbiological aspect is the only thing missing from your article.

SUMMURY:

• I don't quite understand the second sentence: "The literature lacks… stability studies." Could you clarify your point?

• Usually, we don't start a sentence with "pH" but rather "The pH…" or something similar.

INTRODUCTION

• Page 3, line 49. You didn't also conduct the stability study in syringes? Wouldn't that have been relevant?

• Page 3, line 64: Why not use PVC bags anyway? They are the most commonly used for all treatments in North America. But you explain later that this isn't the case for chemotherapy in Europe…

METHODS

• Page 4, lines 73 to 77: Shouldn't the lot numbers of each ingredient be included at a minimum?

• Page 5, line 107: Is it normal to have a packet of "X"s after "PF1"?

• Page 5, line 118: What exactly is a "sale assay"?

RESULTS

• Page 8, line 198: Is it normal for the bottom right-hand cell in the table to be empty?

• Page 9, lines 207, 208, and 216: Why reuse the same numbering, DEG1 and DEG2? Are we still talking about the same data? Or should the numbering be sequential for clarity?

• Page 9, line 219: So, is using an amber bag mandatory?

• Page 10, line 234: Shouldn't the "V" in the temperature line represent the temperature?

• Page 10, lines 234 and 235: You repeat "OC OC" three times.

• Page 10, lines 239 to 242: And what happens with the 5% dextrose?

• Page 10, lines 243 to 245: This key is not below its figure. Here again, do not begin a sentence directly with "pH".

• Page 11, lines 246 to 258: Do these criteria apply to both sodium chloride and 5% dextrose?

• Page 11, lines 253 to 256: This key is not below its figure.

DISCUSSION

• Page 12, line 283: Usually, we avoid referring to "our study" and instead use "the present study," as you have done elsewhere.

• Page 12, line 287: Is it "g/ml" or mg/ml?

• Page 13, line 313: Here again, we avoid referring to "our study" and instead use "the present study," as you have done elsewhere.

CONCLUSION

Correct in itself, very judicious.

REFERENCES:

Correct

APPENDICES:

Correct

**********

what does this mean?). If published, this will include your full peer review and any attached files.

If you choose “no”, your identity will remain anonymous but your review may still be made public.

**Do you want your identity to be public for this peer review?**  For information about this choice, including consent withdrawal, please see our Privacy Policy

Reviewer #1: **Yes:** Damien Lannoy

Reviewer #2: **Yes:** Zribi Kaouther

Reviewer #3: No

**********

[NOTE: If reviewer comments were submitted as an attachment file, they will be attached to this email and accessible via the submission site. Please log into your account, locate the manuscript record, and check for the action link "View Attachments". If this link does not appear, there are no attachment files.]

To ensure your figures meet our technical requirements, please review our figure guidelines: https://journals.plos.org/plosone/s/figures

You may also use PLOS’s free figure tool, NAAS, to help you prepare publication quality figures: https://journals.plos.org/plosone/s/figures#loc-tools-for-figure-preparation.

NAAS will assess whether your figures meet our technical requirements by comparing each figure against our figure specifications.

Attachment Submitted filename: review idarubicin plos one final.docx

Attachment Submitted filename: Commentaire PONE.docx

# Author comment: Author response to Decision Letter 1

**DOI:** 10.1371/journal.pone.0356906.r002

27 Jul 2026

As recommended, all responses to the editor and reviewers are included in the attached document titled “Response to Editor and Reviewers—PLoS One Final.”

Attachment Submitted filename: Response to editor and reviewers-Plos One Final.docx

# Peer review: Decision Letter 1

**DOI:** 10.1371/journal.pone.0356906.r003

**Authors:** Christophe Curti

10 Aug 2026

One-month physicochemical stability study of idarubicin prepared in polyolefin-type bags for the treatment of hematological diseases.

PONE-D-26-07865R1

Dear Dr. Bougéon,

We’re pleased to inform you that your manuscript has been judged scientifically suitable for publication and will be formally accepted for publication once it meets all outstanding technical requirements.

Within one week, you’ll receive an e-mail detailing the required amendments. When these have been addressed, you’ll receive a formal acceptance letter and your manuscript will be scheduled for publication.

An invoice will be generated when your article is formally accepted. Please note, if your institution has a publishing partnership with PLOS and your article meets the relevant criteria, all or part of your publication costs will be covered. Please make sure your user information is up-to-date by logging into Editorial Manager at Editorial Manager® and clicking the ‘Update My Information' link at the top of the page. For questions related to billing, please contact billing support.

If your institution or institutions have a press office, please notify them about your upcoming paper to help maximize its impact. If they’ll be preparing press materials, please inform our press team as soon as possible -- no later than 48 hours after receiving the formal acceptance. Your manuscript will remain under strict press embargo until 2 pm Eastern Time on the date of publication. For more information, please contact onepress@plos.org.

Kind regards,

Christophe Curti

Academic Editor

PLOS One

Additional Editor Comments (optional):

Dear Dr Bougéon,

I have read your point-to-point answer to reviewer reports and the additional experiments conducted.

Your manuscript is well-written and scientifically significant. In my opinion, it can be published in PlosOne without any other revision.

I only have a small remark (which does not justify a minor revision, as your manuscript has a sufficient quality to be published).

I recommended you to use RRTs, but you did not answered to this point. This is not a major point, but it will be useful for the readers (for method transferability).

RRTs are used in Pharma analyses for forced degradation studies and to characterize impurities (see USP and EP monographs, all the impurities are described with their RRTs, and not with their RTs).

RRT is calculated as the ratio of the retention time of a degradation product and the retention time of the studied API (in your work, the idarubicin).

You used the peak purity, which is sufficient for a publication, but for further works, I recommend you to add RRTs (to help the reader to reproduce your experiments).

With my best regards,

Reviewers' comments:

Reviewer's Responses to Questions

**Comments to the Author**

Reviewer #2: All comments have been addressed

Reviewer #3: (No Response)

**********

2. Is the manuscript technically sound, and do the data support the conclusions?

Reviewer #2: Yes

Reviewer #3: (No Response)

**********

3. Has the statistical analysis been performed appropriately and rigorously?

Reviewer #2: Yes

Reviewer #3: (No Response)

**********

4. Have the authors made all data underlying the findings in their manuscript fully available?

The PLOS Data policy

Reviewer #2: Yes

Reviewer #3: (No Response)

**********

5. Is the manuscript presented in an intelligible fashion and written in standard English?

Reviewer #2: Yes

Reviewer #3: (No Response)

**********

Reviewer #2: Dear Editor,

All comments have been adressed and the manuscript can be accepted for publication. It will be usefull for scientific community, working on cytotoxic analysis and stability

Reviewer #3: (No Response)

**********

what does this mean?). If published, this will include your full peer review and any attached files.

If you choose “no”, your identity will remain anonymous but your review may still be made public.

**Do you want your identity to be public for this peer review?**  For information about this choice, including consent withdrawal, please see our Privacy Policy

Reviewer #2: **Yes:** Kaouther Zribi

Reviewer #3: No

**********

# Editor report: Acceptance letter

**DOI:** 10.1371/journal.pone.0356906.r004

**Authors:** Christophe Curti

PONE-D-26-07865R1

PLOS One

Dear Dr. Bouguéon,

I'm pleased to inform you that your manuscript has been deemed suitable for publication in PLOS One. Congratulations! Your manuscript is now being handed over to our production team.

At this stage, our production department will prepare your paper for publication. This includes ensuring the following:

* All references, tables, and figures are properly cited

* All relevant supporting information is included in the manuscript submission,

* There are no issues that prevent the paper from being properly typeset

You will receive further instructions from the production team, including instructions on how to review your proof when it is ready. Please keep in mind that we are working through a large volume of accepted articles, so please give us a few days to review your paper and let you know the next and final steps.

Lastly, if your institution or institutions have a press office, please let them know about your upcoming paper now to help maximize its impact. If they'll be preparing press materials, please inform our press team within the next 48 hours. Your manuscript will remain under strict press embargo until 2 pm Eastern Time on the date of publication. For more information, please contact onepress@plos.org.

You will receive an invoice from PLOS for your publication fee after your manuscript has reached the completed accept phase. If you receive an email requesting payment before acceptance or for any other service, this may be a phishing scheme. Learn how to identify phishing emails and protect your accounts at https://explore.plos.org/phishing.

If we can help with anything else, please email us at customercare@plos.org.

Thank you for submitting your work to PLOS One and supporting open access.

Kind regards,

PLOS ONE Editorial Office Staff

on behalf of

Professor Christophe Curti

Academic Editor

PLOS One
